# Narrative review: The role of circadian rhythm on sports performance, hormonal regulation, immune system function, and injury prevention in athletes

**DOI:** 10.1016/j.heliyon.2023.e19636

**Published:** 2023-09-01

**Authors:** Hadi Nobari, Somayeh Azarian, Saber Saedmocheshi, Pablo Valdés-Badilla, Tomás García Calvo

**Affiliations:** a Faculty of Sport Sciences, University of Extremadura, 10003, Cáceres, Spain; b Department of Exercise Physiology, Faculty of Educational Sciences and Psychology, University of Mohaghegh Ardabili, Ardabil, 56199-11367, Iran; c Department of Physical Education and Sport Sciences, Faculty of Humanities and Social Sciences, University of Kurdistan, Sanandaj, 66177-15175, Iran; d Department of Physical Activity Sciences, Faculty of Education Sciences, Universidad Católica del Maule, Talca, 3530000, Chile; e Sports Coach Career, School of Education, Universidad Viña del Mar, Viña del Mar, 2520000, Chile

**Keywords:** Circadian rhythm, Exercise performance, Hormonal homeostasis, Nutrition, Fatigue

## Abstract

**Objectives:**

This study was a narrative review of the importance of circadian rhythm (CR), describes the underlying mechanisms of CR in sports performance, emphasizes the reciprocal link between CR, endocrine homeostasis and sex differences, and the unique role of the circadian clock in immune system function and coordination.

**Method:**

As a narrative review study, a comprehensive search was conducted in PubMed, Scopus, and Web of Science (core collection) databases using the keywords “circadian rhythm”, “sports performance”, “hormonal regulation”, “immune system”, and “injury prevention”. Inclusion criteria were studies published in English and peer-reviewed journals until July 2023. Studies that examined the role of CR in sports performance, hormonal status, immune system function, and injury prevention in athletes were selected for review.

**Results:**

CR is followed by almost all physiological and biochemical activities in the human body. In humans, the superchiasmatic nucleus controls many daily biorhythms under solar time, including the sleep-wake cycle. A body of literature indicates that the peak performance of essential indicators of sports performance is primarily in the afternoon hours, and the evening of actions occurs roughly at the peak of core body temperature. Recent studies have demonstrated that the time of day that exercise is performed affects the achievement of good physical performance. This review also shows various biomarkers of cellular damage in weariness and the underlying mechanisms of diurnal fluctuations. According to the clock, CR can be synchronized with photonic and non-photonic stimuli (i.e., temperature, physical activity, and food intake), and feeding patterns and diet changes can affect CR and redox markers. It also emphasizes the reciprocal links between CR and endocrine homeostasis, the specific role of the circadian clock in coordinating immune system function, and the relationship between circadian clocks and sex differences.

**Conclusion:**

The interaction between insufficient sleep and time of day on performance has been established in this study because it is crucial to balance training, recovery, and sleep duration to attain optimal sports performance.

## 1 Background

Circadian rhythms (CR) are daily shifts in behaviour and biological activity brought on by an organism's natural capacity to synchronise with its environment's 24-h cycle of light and darkness. These rhythms come from a biological clock inside the body, which regulates many elements of physiology in humans, such as the sleep cycle, daily variations in blood pressure, and body temperature, among others [1]. Numerous studies have been conducted on CR in human physical performance [[2], [3], [4]]. Previous studies have widely suggested that large motor units, including during exercise performance, are directly related to a clear CR [[5], [6], [7]]. As a result, they showed interest in figuring out the reasons behind variations in exercise performance throughout the day. The primary circadian pacemaker in humans is the suprachiasmatic nucleus (SCN). Direct solar cycle information from the retina is received by the SCN, which is found in the hypothalamus [8]. The SCN coordinates daily biological rhythms (such as hormone production, temperature fluctuations, and neuronal activation) following solar time and the sleep-wake cycle using this information transmitted via the retinohypothalamic pathway. Many habits and behaviors are governed by these biological systems' rhythmic oscillations, impacting our daily activities [9]. Studies have shown that sports performance reaches its peak by being in the best position during the day. At this time, the person will have their best mental performance, reaction time, central temperature performance and improved muscle performance. The hormonal response to the CR is different, and their different daily levels can affect sports performance. For example, cortisol and testosterone levels peak in the morning but slowly decrease during the day and increase during the first few hours of sleep [10]. These hormones can affect athletic performance and lead to improved or impaired performance [11]. Finally, this narrative review aims to provide a comprehensive overview of the role of CR in optimal performance, hormonal status, immune system function, and avoidance of injury in athletes. Through a narrative synthesis of existing literature, we describe the underlying mechanisms of CR in sports performance and the reciprocal link between CR, endocrine homeostasis, and sex differences. We also emphasize the unique role of the circadian clock in coordinating immune system function and its impact on physical performance.

Overall, our narrative review highlights the critical importance of CR in almost all physiological and biochemical activities in the human body. The suprachiasmatic nucleus controls many daily biorhythms under solar time, including the sleep-wake cycle. A body of literature indicates that the peak performance of essential indicators of sports performance is primarily in the afternoon hours, and the evening of actions occurs roughly at the peak of core body temperature. Recent studies have demonstrated that the time of day that exercise is performed affects the achievement of good physical performance [12,13]. This review also shows various biomarkers of cellular damage in weariness and the underlying mechanisms of diurnal fluctuations. According to the clock, CR can be synchronized with photonic and non-photonic stimuli (i.e., temperature, physical activity, and food intake), and feeding patterns and diet changes can affect CR and redox markers [14].

Our narrative review highlights the reciprocal links between CR and endocrine homeostasis, emphasizing the importance of a balanced approach to training, recovery, and sleep duration to attain optimal sports performance. Additionally, we discuss the relationship between circadian clocks and sex differences, which highlights the need for sex-specific approaches to optimizing CR in athletes.

### 1.1 Method

The search strategy of this narrative review was developed following the Preferred Reporting Items for Systematic Reviews and Meta-Analyses [15] guidelines. The following databases were searched: PubMed, Scopus, and Web of Science (core collection).

The search strategy included keywords related to “circadian rhythm”, “sports performance”, “hormonal regulation”, “immune system”, and “injury prevention”. These terms were clustered according to the PICO scheme (population, phenomenon of interest, context) to ensure a comprehensive and specific search strategy. The inclusion criteria for articles were.

• Studies published in peer-reviewed journals in the English language.
• Studies published up to July 2023.
• Studies examined the role of CR in sports performance, hormonal regulation, immune system function, and injury prevention in athletes.

The studies were reviewed and synthesized narratively, focusing on the importance of CR in almost all physiological and biochemical activities in the human body. The underlying mechanisms of CR in sports performance were described, emphasizing the reciprocal link between CR, endocrine homeostasis, and sex differences. The unique role of the circadian clock in coordinating immune system function and its impact on physical performance were also discussed.

In summary, we followed a systematic review approach, combining some elements for accuracy and clarity, to create a more rigorous scientific structure for this narrative review.

### 1.2 Circadian rhythm

The phrase “CR” is of Latin origin and refers to oscillations controlled and timed to the environment's natural light cycle for around 24 h, or more precisely, 24.5 h [1,16]. The SCN of the hypothalamus, which receives light and dark signals directly via retinohypothalamic pathways, houses that internal rhythm generator known as the “biological clock.” A central circadian clock processes and transmits information from the outside to the peripheral clocks of various tissues and cells, whose functions may be simultaneous or independent [17]. The pineal gland in the SCN mediates the circadian melatonin cycle, which is predominantly regulated by the light-dark process [2]. This cell-autonomous rhythm is controlled by a network of intricate negative transcription-based feedback loops at the molecular level [18]. The substantial independence of environmental clocks is the sole explanation for many contradictory research findings. These outside signals, also known as natural time synchronizers, are called “zeitgeber” in German literature because they can “wind” the biological clock, controlling CR. Plasma cortisol and plasma melatonin, which, like the precursor of serotonin, are crucial for sleep regulation, are just two of the physiological rhythms affected by light as the primary determinant in addition to the transmission through the retina-hypothalamus circuit [19]. Other than light, zeitgebers include mealtime, physical activity, and various social and psychological aspects. Through the primary biological clock, these zeitgebers impact environmental oscillators, which control numerous critical physiological processes involved in metabolism. Body temperature is thought to improve several physiological rhythms that control significant metabolic variables, serving as the “gold standard” or primary biological marker of the human CR [20].

### 1.3 The importance of circadian rhythm in athletes

Finding elements that boost athletic performance is always of utmost importance in sports. Both professional and amateur athletes can improve their performance if they want to. We live in a “community of performance” where being ranked above everyone else is essential to teaching children fundamental sports values like competitiveness, effort, teamwork, fair play, and maintaining good health. Numerous studies confirm that afternoon exercise increases athletic performance in both professional and amateur players. Performance has improved due to the integration of physiological, psychological, and metabolic rhythms. Together with cardiovascular processes that also follow a circadian pattern, these indicators peak in the early afternoon [21,22]. In particular, it has been calculated that body temperature is 0.9 °C higher in the afternoon [23]. It promotes carbs rather than fats as an energy source and strengthens the actin-myosin cross-links in muscle [24,25]. As a result, even if the mechanism is not entirely understood, afternoon training or physical activity encourages the best muscular performance and enhances muscle development [26]. The greater release of calcium from the sarcoplasmic reticulum, which increases the binding of this ion to actin-myosin and generates the highest ATPase activity of myosin, appears to be the cause of the higher force levels [8,24,25]. While anaerobic performance is well understood, with morning troughs and evening peaks, this is less so for aerobic performance, where the circumstances are murkier, and the findings contradict each other. But it is important to remember that regular training affects performance peaks significantly, especially in the morning. It also improves performance peaks and broadens the range of daily changes at the neuromuscular level when done in the afternoon gives [27].

### 1.4 The importance of circadian rhythm in performance

Early research publications discussed the connection between CR and numerous physiological processes involved in athletic performance, including motor and psychomotor skills, perceptual, and cognitive functions [28]. The circadian clock is controlled to synchronise biological cycles with the environment, incredibly light and physical activity. As previously stated, the primary biological indicator of human CR is thought to be body temperature. Increased body temperature can encourage action and myosin mechanics in musculoskeletal structures, which may improve physical performance through improved skeletal muscle contractile characteristics. Elevated body temperature can also promote the usage of carbohydrates rather than fat as an energy source [29]. According to a large body of research, the body's core temperature is lowest in the morning at 4:30 h. It progressively rises during the day, reaching its highest point around 18:00 h. In the afternoon, a temperature rise is correlated with improvements in coordination, peak reaction time, muscle strength, and cardiovascular efficiency [[30], [31], [32]]. Also peaking in the late afternoon are anaerobic output power and joint flexibility [33]. But when it comes to performance speed, the best outcomes were obtained between 8:30 and 10:30 h in the morning [34]. The reason why athletes perform worse in the morning is that their glycogen stores have been depleted from overnight fasting, their joints have become stiff from periods of rest and sleep, and their muscles have not warmed up as much as they should be compared to the rest of the day's activities, and they are not yet awake enough to keep up their level of action [7]. In a study [35], investigated the effect of fasting during the holy month of Ramadan on the CR of glucose levels in 11 good endurance runners during 24 h in the third and fourth weeks of the holy month of Ramadan. Blood glucose peaked at 20:00 and 04:00 h, which is directly related to food consumption (20:00 h iftar, 04:00 h early morning) and the nadir level was recorded at 16:00 h, which directly affected. With the duration of fasting (12 h from the last meal) Skin temperature, HR, and MAP were directly controlled by physical activity or rest. HR and MAP peaked at 18:00 h, coinciding with the athletes' training time. Also, the decrease in skin temperature in the initial stage of exercise was recorded at 18:00 h and its peak at 12:00 h [36].

The preference for nocturnal or diurnal activities is another significant psychological aspect to consider while researching CR in exercise performance and daily changes in physiological systems. The idea that some persons prefer daily activities while others prefer nocturnal activities has long been acknowledged by Kleitman [37]. Different physiological rhythms, such as sleep-wake cycles, biorhythms (core temperature and hormones), sleep inertia, rhythms of food intake, and maximal oxygen consumption, have been demonstrated to reflect individual differences in time preference (referred to as “chronotype”). It changes when someone workout [38,39].

Given the great range of morning to evening preferences, it is thought that this characteristic reveals an essential capacity for or incapacity to react to various circadian systems. Seven additional variables were also found, which may help explain the CR in exercise performance and may perhaps contribute to the morning performance deficit. Nutritional status varies from morning to evening, flexibility is reduced in the morning, there isn't enough time to recover from sleep inertia, training times are preferred, the amount of time between test sessions varies, each person's physiological response is different, and motivation and expectation's effect are all factors [40,41].

The androgen testosterone (T) effects on muscle strength and exercise adaption are well known [42]. Maintaining anabolism by encouraging protein synthesis in the muscular system is one of T's primary roles [43]. Evidence by Kvorning et al. suggested the significance of this hormone in muscle adaptation by showing that inhibition of endogenous T impaired strength adaptation in healthy male subjects [44]. When things are every day, the circadian profile of T shows a morning peak before gradually declining during the day [10]. Contrarily, glucocorticoid cortisol (C) is frequently employed as a marker of both physiological and psychological stress. It has been demonstrated that a sustained rise in C inhibits the neuromuscular system. According to Ref. [45]., there is a link between poor physical performance and steady rises in salivary C levels. Similar to T, the circadian profile of C peaks in the morning, gradually declines throughout the day, and then rises during the first few hours of sleep [10,46]. Although both variables have an inverse CR pattern, they may still be linked because both steroid hormones and exercise performance substantially affect exercise adaptation [11].

Effects of circadian rhythm on fatigue and non-functional overtraining and decision-making in exercise conditions.

Performance issues are caused by muscle exhaustion [47,48]. The cause of this condition is typically connected to central or peripheral weariness [49,50]. While main tiredness refers to a loss in muscular force brought on by a decline in motor neuron output and/or motor control, peripheral fatigue describes biochemical and ionic changes at the muscle surface that affect the contraction process and/or excitation-contraction coupling [51]. A reduction in the maximum capacity of muscular force production as a result of exercise is known as muscle fatigue [52]. Because short-term maximal exercise performance depends on the time of day, and biomarker variations can impact performance.

The antioxidant system's chronobiological components have received much attention recently. The morning had higher lipid peroxidation levels, total antioxidant capacity, and particular enzyme activity than the evening [53,54]. Physiological levels of melatonin have been found to contribute to the antioxidant capacity of whole human serum, even though the cause of these variations is yet unknown [55]. This hormone has a crucial role as an antioxidant [56]. Lactate levels following the Wingate test were higher in the evening than in the morning, according to research by Hamouda et al. [57].

Interestingly, Racinais et al. found that lactate plasma levels were higher in the evening than in the morning during repeated running activity, which may help to explain why there is more muscle exhaustion in the evening [33]. Given that catecholamines respond to exercise in a manner that is remarkably similar to that of lactase, the difference between the lactate response to exercise in the morning and the evening may be attributed to increased catecholamine activity [58]. Also, according to Dalton et al. diurnal fluctuations in core temperature may impact lactate production during exercise [59]. Moreover, Hamuda et al. demonstrated that tongue temperature and plasma levels of muscle damage enzymes were higher at 17:00 than at 07:00 a.m. [60]. Peak oral temperature cycles are connected with acrophases of these enzymes as well as immunological activities, which could account for alterations in muscular strength during the day [61]. Due to their greater resting values and higher initial power output during short-term maximal exercises in the evening, these enzymes may respond to exercise more strongly in the evening than in the morning. It is also possible that afternoon short-term maximum activity causes muscle fatigue because of greater levels of Homocysteine and other muscle damage indicators and reduces antioxidant status at rest during this time of day [57,60]. Similar to this, Hamouda et al. demonstrated that lactate and glucose responses to the yo-yo intermittent recovery test were higher at night than in the morning, suggesting that anaerobic energy production may play a more prominent role at night (i.e., higher mobility of glucose metabolism at this time of day) [62]. Therefore, diurnal differences in lactate responses have been found after mild and vigorous aerobic exercise, indicating higher anaerobic metabolic activity and muscle fatigue in the evening compared to the morning [63,64].

It is essential to balance the maximum acceptable training stimulus and sufficient recovery to attain optimal athletic performance and competitive fitness. Because of its physiological and psychological restorative benefits, sleep is a crucial component of an athlete's rehabilitation [65,66]. A typical complaint among overworked and/or over-trained athletes is having trouble sleeping [66]. Sleep metrics may be used as markers of overtraining and/or overreaching, regardless of whether sleep decrease is a cause or effect of overtraining and/or overcommitted. When training and recovery are not balanced, overtraining happens [67]. Short recuperation coupled with excessive training volume or intensity accumulation can result in “NFO” and, ultimately, overtraining syndrome, impairing sports performance [68,69]. The recuperation from exercise, both physically and mentally, is said to depend on getting enough sleep [70,71]. And it's thought to be the best athletic recovery technique accessible [72].

Given that a lack of sleep has a direct impact on immune system health [73], when an overtrained or overcommitted athlete performs poorly, this can cause them to experience more stress, anxiety, and depression, all of which can negatively affect their ability to sleep [74,75]. Similar to how lack of sleep can affect mood and heighten stress and anxiety [76]. It is challenging to ascertain the direction of the linkages between sleep, immunological function, stress, anxiety, and mood and how these may eventually result in overtraining or undertraining due to these interrelationships. These findings imply that sleep deprivation can come from overtraining or overreaching or cause these conditions [77]. However, the amount of time an athlete can spend in bed may be impacted by changes in training and competition schedules that frequently accompany greater training loads. It is well-known how training and competition schedules affect athletes' sleep. For instance, early training and competition hours demonstrated to shorten athletes' sleep duration and heighten degrees of pre-exercise weariness [[78], [79], [80]].

**Fig. 1:**
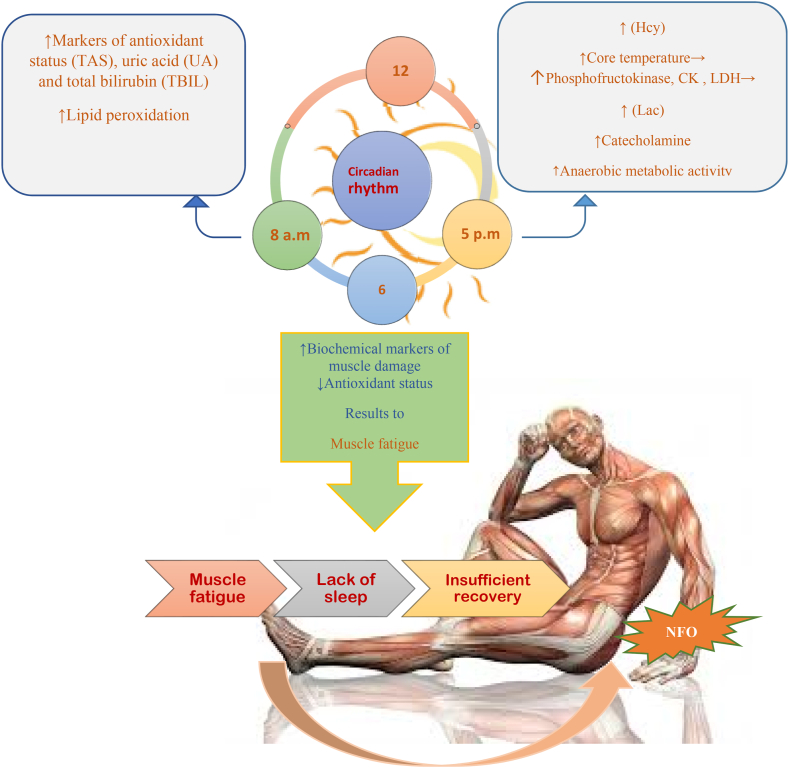
The influencing factors of circadian rhythm in fatigue and non-functional overtraining [82]. Acronyms: Homocysteine (Hcy); Blood Lactate (Lac); Total antioxidant status (TAS); uric acid (UA) and total bilirubin (TBIL); ↑ increase, ↓ decrease.

Typically, compared to daytime sports, nighttime games were linked to later bedtimes, less time in bed, and less overall sleep in a study of Australian rules football players [81]. Therefore, while developing training plans, physicians should consider approaching competition schedules and the effects of training schedules on sleep quality and exhaustion levels [78]. Athletes' opportunities for sleep may be restricted by poorly constructed training plans, which could impede recuperation between workouts. Fig. 1 provides a brief overview of the CR's affecting elements on fatigue and athletic performance.

### 1.5 The importance of circadian rhythm in hormonal status

Periodic changes can be seen in hormonal homeostasis. It is now understood that CR and endocrine rhythms are tightly related, and the internal clock significantly affects how the body responds to environmental influences. Other hormones have been found to fluctuate during the day, but melatonin, cortisol, gonadal steroids, prolactin, thyroid hormone, and growth hormone are among the best (growth hormone) [83]. The release of nutrient-sensing hormones, including insulin, leptin, ghrelin, and adiponectin, is controlled, at least partly, by environmental cues like mealtime and light-dark cycles.

GH levels rise while you sleep and peak right after you fall asleep [11]. CR and IGF-1 levels may be connected through interacting mechanisms. Recently, it was discovered that rats fed ad libitum showed CR in their levels of circulating/hepatic IGF-1. IGF-1 levels were greater during the day and lowered in the liver at night. At the same time, they were higher during the day and reduced during the night in the serum [84]. In a prior study, slow wave sleep (SWS) had much higher growth hormone levels than stages 1 and 2 and rapid eye movement (REM) sleep, which was evaluated during sleep every 30 s [85]. GH is intermittently released when you're sleeping, which may be connected to the cyclical nature of SWS [86]. Compared to healthy persons, patients with post-traumatic stress disorder had reduced amounts of nocturnal GH in their plasma [87]. Children with GH insufficiency receiving growth hormone replacement therapy experienced increased slow Electroencephalography (EEG) oscillations [88].

There is a noticeable diurnal pattern in melatonin. Studies using fixed routine protocols and induced asynchrony show that melatonin levels are higher during the biological night than during the day [89,90]. From the SCN, the paraventricular nucleus (PVN), superior cervical ganglion, and pineal gland are all included in the melatonin secretion pathway [91]. Melatonin has a crucial role in controlling human sleep. Transdermal melatonin or sustained-release melatonin administration enhances sleep maintenance, lengthens total sleep time, and decreases sleep latency [92,93]. Melatonin supplementation raises EEG sleep spindle frequency [93]. Beta-blockers suppress melatonin production. Total wake and sleep time increased in patients taking atenolol and melatonin [94]. In one study using people with cervical spinal cord injury, melatonin production was impaired, and sleep efficiency improved compared to the control group with normal melatonin levels [95]. In another study, where healthy subjects received exogenous melatonin, the average sleep efficiency of healthy subjects improved. When endogenous melatonin was present during the circadian night, it increased by 88%. The beginning of sleep or core body temperature was unaffected by melatonin. The fraction of SWS or REM sleep was unaffected by the melatonin impact, which persisted throughout the course of the study [96]. Also, melatonin has a chronobiotic effect and can help maintain a healthy sleep-wake cycle [97,98].

**Fig. 2:**
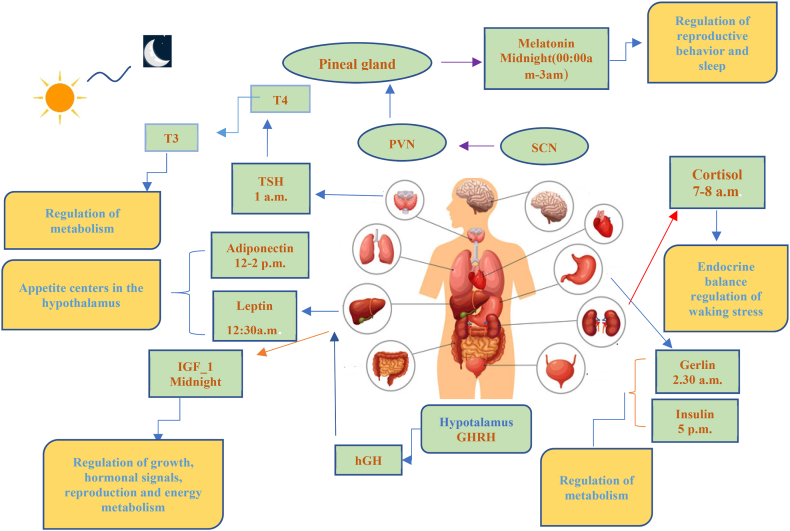
The time of day at which circulating levels of key endocrine factors peak in humans. Acronyms: suprachiasmatic nucleus (SCN); paraventricular nucleus (PVN); thyroid-stimulating, hormone (TSH); insulin-like growth factor 1 (IGF-1); growth hormone receptor [117]; Growth hormone-releasing hormone (GHRH).

Thyroid-stimulating hormone (TSH) concentrations attained their peak and minimum using a set routine technique at biological midnight and biological midday, respectively [99,100]. CR was not linked to total triiodothyronine (T3) or thyroxine (T4) concentrations [100]. TSH levels and SWS are negatively correlated [101,102]. There is diurnal rhythmicity in cortisol. At biological midnight, its level quickly increases, reaching a high at biological morning [103,104]. An ultradian CR drives the pulsatile release of cortisol during the course of 24 h. Gonadotropin-releasing hormone's pulsatile release inhibits receptor desensitisation [105,106]. At the middle of this range of rhythm, regulation is the SCN. The dorsal nucleus of the hypothalamus (DMH), the PVN, and the paracellular nerve that triggers corticotropin-releasing hormone (CRH) are all parts of the hormonal system that underlie this control [107]. The spinal cord is the final link in the neuronal circuit that regulates cortisol levels, which extends from the SCN to the PVN and finally to the adrenal cortex [107]. During SWS, cortisol levels decline. In addition, there is proof that reduced cortisol levels and SWS are related in time. Intravenously infusing cortisol increased SWS and decreased REM sleep. According to Steiger's research [108]. Leptin and ghrelin increase and inhibit food intake [109,110]. Before frequent meals, ghrelin levels rise and fall afterwards [111,112]. A few research studies examined the association between sleep and hormone levels [113]. After intravenous ghrelin injection, it was shown that growth hormone levels and the SWS ratio increased, whereas REM sleep decreased [114]. SWS increased, and REM sleep decreased after leptin infusion in research on rodents [115]. The proportions of stage 2 and SWS sleep were enhanced, and those of stage 1 and REM sleep decreased in older males who had been given ghrelin [116]. Alternatively, it has been noted that ghrelin levels rise during the first stages of sleep and fall following sleep deprivation [113]. However, the stories of ghrelin and sleep stage were not significantly correlated in another investigation [114]. Leptin levels rose over the biological night and peaked in the physical morning, according to one study [115]. However, CR did not cause any changes in the levels of leptin, according to Scheer et al. [105]. Fig. 2 summarizes the hormone variations throughout a 24-h period.

### 1.6 The importance of circadian rhythm in immune system function

Perhaps the most evident organismal reaction to infection is the immunological response, and mounting evidence points to the circadian clock as a critical regulator of immune defence. The circadian system is primarily responsible for controlling CR, which are endogenous processes with an oscillating pattern that follow a daily cycle [116]. The SCN synchronises time with day/night cycles by detecting and integrating light signals. It externally sets signals that are then used to synchronise environmental clocks. Circadian oscillations in cellular processes, including gene expression, protein translation, intracellular signaling, metabolism, and many cell type-specific functions, are maintained at the cellular level by a molecular clock mechanism [118]. This is because different types of immune cells have molecular clocks, and the immune and circadian systems interact in various ways to control the timing of numerous processes that control immune surveillance and response to infection [119,120]. Nearly all aspects of innate and adaptive immunity show a fluctuating daily pattern, including immune cell traffic (signaling) and circulating humoral components, inflammatory processes, response to infection, cytokine expression, chemokines and receptors for detection, signaling, among others [120,121]. Interestingly, rhythmicity's essential mechanisms, including transcriptional variability, occur in the chromatin fibre [118,122].

**Fig. 3:**
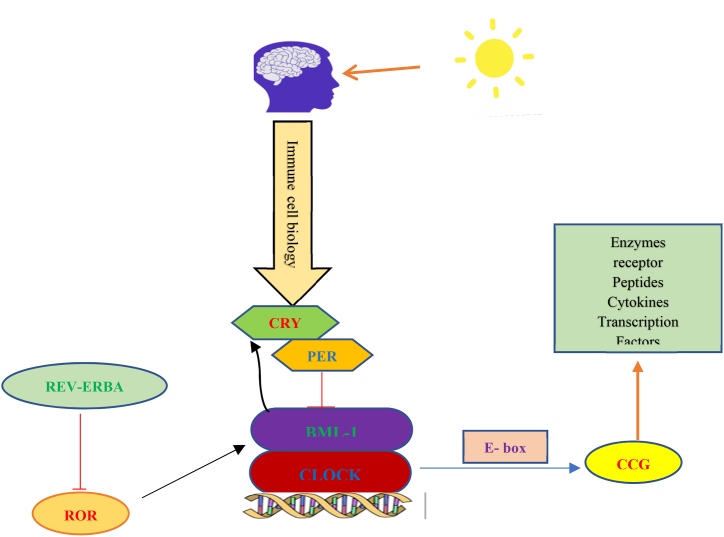
The Circadian system and molecular clock mechanisms central clock cells in the SCN and peripheral clocks in many other tissues, including the immune system, have a clock based on self-regulating feedback loops. Acronyms: heterodimer composed of the proteins BMAL1and CLOCK (BMAL1/CLOCK); clock gene products Per, Cry (also known as REV-ERBα/β, and RORα–γ); E-boxes (ROR-responsive elements); clock-controlled genes (CCG).

Numerous studies have shown that the immune response is strictly regulated by the circadian clock, even though hundreds of genes are transcribed daily, most of which regulate vital activities to maintain homeostasis [121]. CR interferes with immunity, as seen by ongoing changes in immune cell traffic in the blood. For instance, in mice and people, the number of circulating neutrophils, monocytes, and lymphocytes changes throughout the day [[123], [124], [125]]. Interestingly, under homeostatic or inflammatory settings, circadian-controlled brain signals affect leukocyte migration to tissues [126]. See Fig. 3.

How does circadian rhythm help to prevent non-contact injury?

Photonic and nonphotonic stimuli can synchronise the circadian clock (e.g., temperature, physical activity and food intake). Since physical activity significantly disrupts cells, tissues, and organs and challenges overall body homeostasis [127]. For healthy and practical reasons, existing ideas are starting to investigate the connection between exercise and CR. According to a substantial body of research [117], rodents' circadian systems are affected by training, and growing human evidence suggests that movement can also cause phase-shifting effects that may be chronotype-dependent [128]. Muscle clocks and other peripheral and central clocks appear strongly entrainable by exercise. For instance, male rugby players exhibit considerably higher mean central clock gene expression (BMAL1, ROR-, CRY1, PER2, PER1, and NR1D1) than sedentary males [129,130].

Body temperature, which can have various regulatory effects on neuromuscular and metabolic activity and seems to peak in the late afternoon, is a significant factor in these performance variations. The onset of weariness may vary when the same action is performed at various times throughout the diurnal cycle, particularly during more prolonged bouts of endurance-type exercise. It may be explained by thermoregulation during training appears to follow a CR [131]. Clock-driven oscillations in metabolic regulation, such as glucose metabolism requiring less oxygen consumption, lower heart rate, and lower perceived exertion, have been linked to changes in human exercise performance (late vs. early better) [132]. Although it is not an entirely novel idea, this association between exercise capacity and the molecular clock in competitive and elite sports situations may be helpful when planning to improve training programs and competitions [133]. Strength training studies have demonstrated that an increase in oxidative stress due to exercise causes a response to muscle injury [134]. The afternoon (from 13:00 to 20:00 p.m.) is when muscle regeneration and repair processes are at their highest. Higher uric acid levels are also associated with an increase in antioxidant capacity, which is mediated by peak catalase and glutathione. Both peroxidase and PM are identified [135]. The highest amounts of HDL, triglycerides and glucose were discovered in the afternoon in the studies that involved strength and endurance training, which showed improvement in performance throughout the day. The peak time for creatine kinase levels, a marker of muscle injury, is likewise in the afternoon. The number of research studies on this subject that discuss the impact of the time of day on physical performance is substantially higher (n = 19) [62].

Coaches and players are more conscious of the value of training time in achieving positive results today. Most studies has suggested that performance is higher in the late afternoon, particularly between 16:30 and 19:00 h [136,137]. While working with weightlifters, some authors, like Ammar et al. report improved performance and decreased perceived effort (RPE) ratings up to 2:00 a.m. More so than the day of the week, the time of day impacts the body's physical response [138], even though performance in the higher morning has occasionally been assessed [139,140]. Injuries caused by sports training in athletes can have different causes. In adolescent athletes, according to the studies, people who slept less than 8 h a night experienced 1.7 times more injuries compared to people who had enough sleep [141]. Also, research has shown that recovery in professionals is at its best during sleep. During NREM sleep, the growth hormone released from the pituitary gland can play an important role in tissue regeneration and restoration. Also, in these conditions, oxygen consumption decreases and the level of protein synthesis and fatty acid transfer increases, which helps recovery [141].

### 1.7 Nutritional considerations for improving athletes' circadian rhythm

Diet significantly impacts CR, with breakfast as the primary coordinator following the most extended fast. Because early morning sympathetic activity triggers the most metabolic processes, this is the time of day when the body needs more energy to go through the rest of the day's activities. However, there is an increase in stomach emptying, which causes a rise in intestinal absorption and glucose tolerance [142]. Athletes should consider the type of diet and the (regular) frequency of their feeding. Numerous “environmental clocks” play a part in the body's circadian control of lipid levels. Because they modify the expression of circadian genes, often known as “clock genes,” which activate or inactivate other factors that induce physiological changes in cells over 24-h periods, fat-rich diets are regarded as powerful chronodisrupters [143]. As a result, high-fat diets disrupt CR and metabolic alterations, which is terrible for athletes' health [36,144].

The relationship between food and the circadian system is called chrono nutrition. It has been proposed that altering the timing and kind of food intake can affect the internal clock [145]. The 5-hydroxytryptophan (5-HT), GABA, orexin, melanin-concentrating hormone, cholinergic, galanin, noradrenaline, and histamine are some the neurotransmitters that are involved in the sleep-wake cycle [146]. As a result, nutritional strategies that impact these neurotransmitters may benefit sleep. Neurotransmitter production and function can be affected by dietary antecedents (for example, serotonin synthesis depends on the availability of the precursor tryptophan in the brain). A system that shares transporters with some large neutral amino acids (LNAA) is used to transport tryptophan over the blood-brain barrier [147]. Tryptophan, a high-carbohydrate/low-protein diet, or lactalbumin, a whey-derived protein, can help raise the blood's tryptophan: LNAA ratio, which is necessary for the transport of tryptophan to the brain [148]. Consuming carbohydrates increases plasma tryptophan concentration [149]. Consuming carbohydrates may enhance the sleep-promoting effects of tryptophan-rich proteins and impact the plasma tryptophan: LNAA ratio [150]. Following a meal high in carbohydrates, insulin, an anabolic substance that also makes it easier for LNAA to be absorbed by muscles, alters the movement of tryptophan over the blood-brain barrier [151]. Through an immediate impact of insulin that improves muscle absorption of LNAA, high glycemic index (GI) carbohydrate consumption raises the ratio of circulating tryptophan to LNAA [152].

Lower carbohydrate intake (24-h recall and structured interview) was substantially (OR = 0.71; 95%CI = 0.55 to 0.92, *p* = 0.01) linked with insomnia symptoms (culture of keeping sleep) across a sizable sample (n = 4452) from the National Health and Nutrition Examination Survey [153]. Consuming a high-carbohydrate dinner (130 g) 45 min before bedtime increased REM and decreased light sleep and alertness compared to eating a low-carbohydrate meal (47 g) or no carbohydrate meal [154]. Further research in athletic groups is necessary to determine the effects of carbohydrate evening meals on sleep and recovery in athletes: Table 1 details the nutritional interventions and their impact on sleep results.

**Table 1:** Changes to sleep following nutritional interventions.

Study	Type- Dosage	consumption timeC	Effect
Lin, 2011 [155]	two kiwifruit	hour before bedtime	↑The property of sleep – ↑ TST
Valtonen, 2005 [156]	100 g or of 500 g(a large dose) normal commercial milk	daily dose	no effect on sleep
Garrido, 2010 [157]	Sour cherry juice	twice a day	↓insomnia
Howatson, 2012 [158]	Sour cherry juice (2 servings of 30 ml concentrate)		↑SE- ↑ TST ↓daytime napping

SE: sleep efficiency, TST: total sleep time, ↑ increase, ↓ decrease.

### 1.8 Supplements for improving athletes' circadian rhythm

Any supplement an athlete chooses to take must be taken safely and efficiently. Athletes should ensure that any supplements they take have undergone drug testing for prohibited substances and that there are independent testing methods, such as informed exercise, educated choice, or additional protection. Athletes should consult a professional sports nutritionist or nutritionist before using any nutritional supplements. By following the best nutritional strategies, exercise adaption and recovery might be improved or hampered [159,160]. Nutrients like carbs (eating an evening meal with a high glycemic index decreases the time it takes for sleep to start), protein (drinking dairy products may lengthen sleep), ethanol (REM sleep is reduced) [161], and caffeine (increases sleep onset delay, shortens total sleep time, and lowers the quality of sleep) [145]. CR can also be influenced by the timing and amount of meals (large portions and/or afternoon meals can potentially impair sleep due to the thermic action of digestion) [150]. Poor sleep can result from calcium intake, which can then result in more calcium being consumed. Because caffeine inhibits adenosine receptors, it makes people more awake and reduces their propensity to fall asleep [145]. Alcohol use is linked to decreased REM sleep, reduced sleep duration, and increased sleep disruption in the second half of the sleep cycle [162].

Melatonin has been shown to help increase sleep quality and circadian components of the sleep-wake cycle, according to Leonardo-Mendonsa et al. [163]. Melatonin increases sleep duration, decreases nocturnal awakenings, decreases nocturnal activity and promotes sleep when given before bed [163,164]. According to Atkinson et al. consuming 5 mg of melatonin in the morning substantially impacted matched individuals' future physical performance [165]. Another study by Qatasi et al. discovered that morning melatonin injection had no negative impact on soccer players' afternoon physical and cognitive performance [166].

Tryptophan is an essential amino acid precursor of serotonin and melatonin, which can cross the blood-brain barrier by competing for transport with other LNAAs [147]. Conversion to serotonin depends on the brain's ability to produce enough precursor, increasing brain Tryptophan when the ratio of free tryptophan to branched-chain amino acids rises. Melatonin is then formed when serotonin is made from tryptophan [147]. Comparing dietary tryptophan to food bars (Food 1: 25 g oil-free pumpkin seed meal and 25 g dextrose, Food 2: 250 mg medicated tryptophan, and Food 3: 50 g [Control]) [167]. Foods 1 and 2 significantly improved sleep efficiency (% time spent in bed asleep; 5.19% and 7.36%) and reduced waking time during the night (19.2% and 22.1%, respectively). And better subjective sleep quality (12.2% and 11.8%), indicating that even relatively low amounts of dietary tryptophan (250 mg) can have an advantageous effect [167].

Melatonin is produced partly by vitamin B12, serotonin is paid in part by vitamin B6's pyridoxine, and tryptophan may be reduced by vitamin B3's niacin [150]. Melatonin is produced in part by vitamin B12, serotonin is produced in part by vitamin B6's pyridoxine, and tryptophan may be reduced by vitamin B3's niacin [150]. Tryptophan is converted to serotonin by pyridoxine and folate, vitamins B9 [152]. Tetrahydrobiopterin, a typical tryptophan-5-hydroxylase enzyme that transforms tryptophan into 5-hydroxytryptamine (5-HT), is increased by the reduced form of folate (5-methyltetrahydrofolate) [152]. The amino acid decarboxylase enzyme, which speeds up the conversion of 5-HT to serotonin, is linked to the role of pyridoxine in the conversion of tryptophan to serotonin [152]. Different cobalamin (vitamin B12) doses have had conflicting effects on sleep-wake rhythm and delayed sleep phase syndrome (significant CR delay) while not affecting sleep duration [162].

A nutritional supplement comprising 5 mg of melatonin, 225 mg of magnesium, and 11.25 mg of zinc significantly (*p* ≤ 0.001) enhanced subjective sleep quality scores, according to a double-blind, placebo-controlled research of senior participants (n = 43). Actigraphy was used to assess the improvement in sleep in the intervention group but not the control group (group difference 6.8; 95% CI = 5.4 to 8.3) and total sleep duration (182.18 min; 95% CI = 204.34 to 160.02) [115]. The synergy of magnesium, zinc, and melatonin was said to be responsible for these benefits; however, it should be highlighted that supplementing with these nutrients will probably only be helpful in circumstances of deficit or insufficiency [168]. Table 2 displays information about complementary therapies and their impact on sleep outcomes.

**Table 2:** Sleep changes following complementary interventions.

Study	Type- Dosage	Consumption time	Effect
Pires, 2001 [169]	Exogenous melatonin (0.3 mg or 1 mg)	6:00 p.m.	↑SOL
Porter, 1981 [154]	Carbohydrate (47 g–130 g)	45 min before bedtime	↓light sleep- ↑ REM
Afaghi, 2007 [149]	Carbohydrates with a high glycemic index (GI).	4 h pre-bed	↓SOL
Hudson, 2005 [167]	oily pumpkin seed powder (25 g)- dextrose(25 g)-medicinal tryptophan(250 mgr)	–	↑SE _ ↓ WASO
Markus, 2005 [170]	Contains milkshakes Lactalbumin (20 g)	Evening snack	↓ morning Sleepiness
Rondanelli, 2011 [168]	food-based supplement (5 mg melatonin, 225 mg magnesium and 11.25 mg zinc)	–	↑TST
Leonardo-Mendonca, 2015 [163]	Melatonin (100 mg)	Before starting sleep for 4 weeks	↑ sleep quality
Lopez-Flores, 2018 [171]	Melatonin (10 mg)	30 min pre-slee*p*	↑sleep quality
Meolie, 2005 [172]	Magnesium oral supplement	Eight weeks 1 h before bedtime	↑sleep quality- ↑ TST

SE: sleep efficiency, SOL: sleep onset latency, TST: total sleep time, ↑ increase, ↓ decrease.

### 1.9 Can circadian rhythm help homeostasis?

This section will describe hormonal changes and the immune system. Also, check the best times of CA, such as showing the right amount of sleep, afternoon nap, and before training. Numerous physiologically regulated processes have been demonstrated to be time-of-day dependent (TOD), and healthy adults at rest have physiological CR that are well understood [173]. Since the antioxidant system is more effective in the morning and the rate of lipid peroxidation is higher in the early evening, it has been well established that in active and healthy individuals, indices of oxidative stress and antioxidant status depend on the time of day [173,174]. Similar research has shown that blood lactate (Lac), alkaline phosphatase (PAL), gamma-glutamyl transpeptidase (GT-g), creatine kinase (CK), lactate dehydrogenase (LDH), alanine aminotransferase [175], aspartate aminotransferase (ASAT), and other biochemical indicators of muscle damage and fatigue depend on the time of day and have significantly higher values in it has already been noted [176,177]. The resting values of white blood cells (WBC) and their subgroups of monocytes (MO), neutrophils (NE), and lymphocytes (LY) are significantly greater in the evening than in the morning in healthy sedentary individuals [173]. In general, the afternoon was when the levels of urea (URE), creatinine (CR), glucose (GLC), total cholesterol (TC), triglyceride (TG), and high-density lipoprotein (HDL) were at their lowest [173,177] (Table 3).

**Table 3:** Physiological effect of sleep on Biomarkers of muscle injury.

Study	biomarker	Physiological effect	sleep stages			
			NSN	PSD		
Mejri, 2015 [178]	CK	CK increasing in post exercise of Yo-Yo intermittent test		PSDEN	PSDBN	
				*		
Mejri, 2015 [178]	Lac	post-exercis → a slight decrease(Lac) → after PSDEN		*		
Knutson, 2007 [179]	plasma GLC levels	early awakening → in greater glycogen depletion - higher plasma GLC levels- increased GLC →by greater insulin resistance		*		
O'Neill, 2011 [180]	RBC and HB	At rest after PSDEN → < PSDBN and baseline		*		
Abedelmalek, 2013 [181]	WBC and GR	the short-term high-intensity exercise after PSDEN→higher WBC and GR→the greater inflammation		*		
Rae, 2017 [182]	interleukin-6 – basophil	during the recovery →remained elevated→ period after sleep deprivation		*		
Mejri, 2015 [178]	urea (URE)	Increased URE→increase in ammonia		*		
Mejri, 2015 [178]	aspartate aminotransferase (AST)	(AST) increased→ More challenge from training for muscles and liver		*		
Mejri, 2015 [178]	creatine kinase (CK)- monocyte (MO)	higher resting		*		
Alzoubi, 2012 [183]	glutathione peroxidase (GPx)	decrease in resting → post-exercise		*		
Savic, 2015 [184]	superoxide dismutase (SOD)	sleep deprivation→ declined SOD		*		

NSN; normal sleep night, PSDEN; partial sleep deprivation at the end of the night, PSDBN; partial sleep deprivation at the beginning of the night.CK, creatine kinase,:WBC, wight blood cell: RBC, red blood cell.

CR regulates nearly all hormones; in humans, the natural endocrine cycle lasts around 25 h; the cycle of sleep, wakefulness, and light-darkness affect the concentration of plasma corticosteroids. According to scientists, blood pressure fluctuates throughout the day depending on the time of day. Both people with normal and high blood pressure have a sharp rise in their blood pressure in the early morning hours [185]. Numerous studies indicate that the minimum threshold is between zero and three in the morning, and the highest threshold is between 12:00 to 18:00 h, because at night people become more sensitive to pain. Because people have a higher pain tolerance towards the end of the day, sedative medicines work less effectively at night to lessen pain than during the day. They are somewhat different physiologically in how they affect the circadian cycle [186].

Two factors are principally in charge of controlling the dynamic process of sleep. Homeostasis for rest and CR. To demonstrate the connection between the circadian clock (an endogenous timing system) and the homeostatic sleep stimulus (sleep pressure or an urge to sleep that builds up during waking), a two-process model of sleep regulation was created [187,188]. The circadian process is governed by a circadian oscillator, whereas the homeostatic process depends on sleep and wakefulness [189]. Homeostatic processes change during awake and sleep, interact with C regardless of insomnia or sleep, and take signals from the environment (such as light) [187,188]. Although the brain's SCN serves as the process's focal point, auxiliary clock systems have been discovered elsewhere over the body [187].

The immunological and endocrine systems are restored by sleep, which helps the body recover from the metabolic and neuronal costs of waking up [190,191]. Sleep is also crucial for learning, memory, and synaptic plasticity, which refers to the ability of synapses to become stronger or weaker over time. Sleep, particularly slow-wave (or N3) sleep in the early hours of the night, increases prolactin secretion while decreasing cortisol and catecholamines' anti-inflammatory effects [190]. Acute sleep deprivation and sleep disruption (short sleep duration or reduced sleep frequency) reduce adaptive immunity, which is linked to a reduced response to vaccination and increased susceptibility to infectious diseases, which is linked to a decreased growth hormone secretion during deep sleep and an increased sympathetic outflow [192].

Lack of sleep is linked to a rise in catabolic hormones and a fall in anabolic hormones, impairing muscle protein synthesis [181] and reducing recovery and training adaptations. There have been reports of sleep problems and inadequate sleep duration among athletes. Polysomnography (PSG) analysis of the sleep habits of 23 professional male ice hockey players revealed an average total sleep time of 6.92 h (95% CI = 6.3–7.5 h) [193]. Like other athletes, South African athletes (n = 890; international n = 183, national n = 474, club n = 233) claimed that sleep was their primary means of recuperation [22]. While a previous study found that 66% of German professional athletes (n = 416) reported having signs of pre-competition insomnia, such as difficulty falling asleep, nightly awakenings, and early final waking times reported [194]. The most significant indicator of injury in adolescent athletes is reported to be 8 h (OR = 2.1; 95% CI = 1.2 to 3.9) [195]. Elite Swedish adolescent athletes were studied as part of the Karolinska Athlete Screening Injury Prevention Study (KASIP), which found that those who slept more than 8 h had a lower risk of injury (OR = 0.39; 95% CI = 0.17 to 0.96) [196].

Surprisingly, little research looked at how much sleep affects sports performance [[186], [187], [188], [189], [190], [191]], the majority of which assessed short-term performance with a sizable anaerobic component and discovered that lack of sleep had no impact on performance [197,198]. However, other studies, many of which assessed endurance performance, have noted lower performance following sleep deficit or recovering from sleep deprivation. The negative impact of sleep loss on endurance performance may be influenced psychologically, such as motivation [199]. Lack of sleep seems to negatively impact evening performance more than morning performance [[199], [200], [201]]. After sleep loss, decreasing CR amplitude probably causes decreased performance in the evening [200,202]. Only a few studies measure performance after total sleep deprivation [199,200], a restriction that would have produced less meaningful outcomes. Regarding relative sleep deprivation, missing the first few hours of the night doesn't seem to affect performance [201,203,204]. However, partial sleep deprivation over multiple days has been shown to have the opposite effect, probably because it increases perceived effort, sleepiness, and weariness [205].

A 30-min nap in the afternoon following a night of relatively little sleep (waking around 3:00) has also been found in one study to enhance running performance in comparison to a no-nap condition speed up by 2–20 m [206]. Napping may improve alertness and motivation for work by lessening the weariness brought on by sleep deprivation [206,207]. Power estimations were not often published in most studies that looked into sleep deprivation. The impact of sleep deprivation may be diminished because of the limited statistical power.

Further evidence that adequate sleep duration is crucial for athletes comes from the associations between napping while sleep-deprived and increased performance [206,208]. If the athlete has enough time to overcome sleep inertia, napping, whether brief or prolonged, involving deep sleep or not, is likely to be beneficial [207]. Table 4 provides specifics regarding the impact of regular sleep on performance.

**Table 4:** Sleep, performance, sleep duration, amount of sleep for adult.

Study	Sleep duration	Performance
Blumert, 2007 [209]	24 h of sleep deprivation	↓physical performance of collegiate weightlifters
Brauer, 2019 [210]	sleepless night	↓ Glycogen storages
Abedelmalek, 2013- Hajsalem 2013 [181]	Relative sleep deprivation (3–4 h)	↓Performance- ↓peak and mean anaerobic power
Mah CD, 2011 [211]	extended sleep (five-seven week) (10 h)	improved →times on sprints ↑free throw ↑3-point shooting accuracy in the basketball player
Schwartz and Simon, 2015 [212]	sleeping time up 1 week (9 h)	improved→ the service accuracy from 36 to 42%
US Department of Health and Human Services, 2015 [213]	Helpful sleep for adults≥7	Optimum health promotion

↑ increase, ↓ decrease.

It is also important to note that the use of electronic media (e.g., smartphones) continues to increase in the modern era [214]. Smartphones, laptops, and e-books equipped with light-emitting diodes (LEDs) that emit significant amounts of short-wavelength light (i.e., “blue light”) [215,216]. These light sources show a spectral peak at around 460 nm [217,218]. Melanopsin is particularly sensitive to light with short wavelengths between 446 nm and 480 nm [219], thus to the light emitted preferentially from LED screens (for example, smart phones) is emitted. Information about light is further relayed via the retinohypothalamic tract to our “internal clock” located in the suprachiasmatic nuclei (SCN) of the hypothalamus. The SCN then projects to the pineal and pituitary glands. The pituitary gland controls and stimulates the secretion of the stress hormone cortisol [220]. About light-induced cortisol changes, a study examining exposure to blue-enriched light (1500 lux) in the evening (23:00 to 24:00 h) showed no immediate effect on cortisol secretion [221], whereas Morning (05:00 to 08:00 h). Exposure to bright light (2000–4500 lux) increases cortisol secretion [222]. However, the lasting effects of exposure to enriched or bright blue light in the evening on morning cortisol levels were not assessed in these studies. It is controlled by the pineal gland and projects back to the SCN [17]. As a result, melatonin strengthens sleep pressure and inhibits the desire to stay awake [17]. Regarding the effect of light, melatonin is strongly affected by exposure to short-wavelength light in the evening. For example, 2 h of exposure to 460 nm light in the late evening (i.e., 21:30 to 23:30 h) significantly suppressed melatonin secretion compared to 540 nm or no light [17]. Consistent with these results, 5 h of LED screen exposure before sleep initially suppressed melatonin secretion, followed by a delayed increase [214].

This allows for the assumption that exposure to evening light also affects sleep physiology, particularly the amount of SWS and slow wave activity (SWA, 0.75–4.5 Hz), respectively. Indeed, two studies reported a reduction in SWS and SWA in the first sleep cycle after participants were exposed to short-wavelength light in the late evening (i.e., between 21:30 to 23:30 h) [215]. These findings suggest that the negative effects of light on sleep may affect behaviour the next morning. Evidence from previous studies shows opposite outcome patterns for wakefulness and sleepiness in the morning after sleep [223,224]. Therefore, short-wavelength light in the previous evening reduced alertness the following morning, because its alerting effects in the evening may interfere with subsequent sleep.

A large body of research well supports the impact of the time of day on performance. Two factors are principally in charge of controlling the dynamic process of sleep. Technical skill-based sports like badminton, tennis, and soccer seem to have an acrophase a little bit earlier in the day (i.e., afternoon) [5,32,225] than muscle power and anaerobic capacity, which appear to peak in the early hours of the morning [[226], [227], [228], [229], [230], [231], [232]]. Technical skills are known to rise in the morning and increasingly need fine motor control [207]. In general, the waking time has a greater impact on cognitive and sensorimotor aspects of diverse processes than on gross motor motions [233]. These findings indicate a relationship between performance, sleep deficiency, and time of day.

### 1.10 Circadian rhythm and gender

Even in infancy, there are subtle sex variations in sleep, but little research has been done in this area [234]. After birth, sex differences in EEG waveforms are seen, and girls spend more time overall around ten months than boys [235]. Sex variations in sleep structure ultimately manifest at this point [236]. Interestingly, baby males establish a coherent sleep cycle a little later than girls at the same age [237].

A change in chronotype characterises the gender difference in sleep that appears during adolescence. Girls begin puberty on average one year earlier than boys do. Girls start to experience a chronotype shift at the same time. So, they begin staying up later than males a year earlier, which releases gonadal hormones. However, girls experience the peak sleep onset delay one year earlier than boys [238]. There also seem to be gender disparities in the rate of chronotype change, in addition to gender differences in the timing of chronotype onset. Boys experience a bigger overall chronotype shift than girls do. Most gonadal hormones are implicated in the chronotype alterations seen in adolescence through a link between the development of secondary sexual characteristics and age-related changes in sleep onset [239,240].

Adult sleep behaviour has been linked to gender variations in several ways. Females typically go to bed sooner than males, stay in bed longer than men, and get more overall sleep [241]. With the start of menopause, which once again impacts the hormonal influence on the circadian and sleep systems, it is interesting to note that female's urge to sleep fades earlier than the male's throughout adulthood [238].

Interestingly, despite getting total sleep than males, females report having worse sleep than males, and their chance of developing insomnia is 41% higher [242]. It is unknown what precisely accounts for this significant difference. In a study that evaluated the EEG activity of insomnia patients and healthy controls, males with insomnia and healthy controls' EEGs were not substantially different. In contrast, the EEG of females with insomnia and healthy controls differed during all NREM sleep stages. This discovery might be connected to the rise in female sleeplessness [243].

In reaction to lack of sleep, the body of knowledge regarding sex differences in human sleep homeostasis and the effects of sleep deprivation is expanding. Because the impact of building up sleep debt differ for males and female, there appears to be a gender variation in how people react to sleep deprivation [234,244]. Sleep debt negatively affects females' health more than males because females build sleep debt more quickly, and its effects are more severe in females [245], as their health is more adversely affected [244]. Because females experience higher slow-wave activity (SWA, also known as activity) during NREM sleep than males, objective sleep measurements reveal sex differences even at baseline [246,247]. The finding that females exhibit a more significant restoration of SWA in response to sleep deprivation than males dovetails with the theory that the S process may differ between the sexes [245]. Female mice exhibit more NREM sleep than male mice for the first 2 h following sleep deprivation, shows that sex differences in homeostatic sleep mechanisms may exist in rodents and humans [248].

### 1.11 Practical applications

Studies on the relationship between circadian principles and immunological function, and hormone balance may demonstrate which of these strategies helps athletes retain performance, in our opinion. The scientific literature that is now available suggests that there is still an opportunity for improvement, but any future research must be well planned to account for confounding variables, including participant chronotype, the influence of other zeitgebers, and under-testing. The promising improvement brought about by supplemental nutrition administration during sleep programs calls for additional parallel research. It's crucial to note that new research could profit from carefully selecting proper techniques for measuring and assessing the effect of proposed interventions on CR. Of course, given that dietary and feeding modifications may have an impact on redox indicators and CR [249]. Using calorie restriction is a good substitute.

NADPH oxidase, an important marker of the formation of oxidative stress, increased after 12 weeks of high-fat feeding in animal models, speeding up the pathogenesis of endothelial dysfunction [250]. Rodents fed a high-fat diet for a further 12 weeks of calorie restriction with and without exercise training, however, demonstrated normalization of NADPH oxidase levels and reversal of the degenerative development of endothelial dysfunction. In obese females, a 10% weight loss after three months of food restriction (energy deficit of 500–1000 kcal/day) increased glutathione reductase [251], but a 20% weight loss after six months of a low-calorie diet was sufficient to boost GPx. Indicating a correlation between enough BMI reduction and decreases in triglyceride, IL-6, and 8-isoprostane levels [252]. In addition, intermittent dietary restriction (20%) caused obese adults' 4-HNE and serum 8-isopropionate levels to decrease while raising their antioxidant concentration [235] significantly. Wycherley et al. demonstrated that although diet and diet combined with aerobic exercise increased oxidative stress and nitric oxide (NO) availability and significantly reduced body weight in obese participants with T2 diabetes, there was no difference between the therapies after 12 weeks [253].

Numerous studies have shown that the quantity and composition of macronutrient consumption might affect postprandial oxidative stress responses. As opposed to ingesting moderate (75 g) and high (150 g) amounts of dextrose, consuming 66 g of fat significantly raised the levels of the oxidative stress markers MDA and H2O2. It's possible that postprandial superoxide production helped to cause oxidative stress [254], perhaps associated with the generation of postprandial superoxide [255]. After consuming 33 g of fat, various outcomes were reported, demonstrating that postprandial oxidative stress responses can be influenced by the source, amount, and distribution of macronutrients [256]. In addition, compared to isocaloric meals with various macronutrient compositions, postprandial triglycerides, TAG, MDA, H2O2, and nitrate/nitrite have been shown to increase [240] significantly. The first study comparing the oxidative stress response brought on by acute bouts of intense exercise with high-fat meals was conducted by McCarthy et al., exercise may have increased antioxidant defences in the study participants, which may have contributed to the non-significant rise in oxidative stress after strenuous exercise [257].

This food adjustment has been demonstrated to extend mice’ lives [258,259]. These results might not apply to all species in any case. A CR diet can also be contrasted with an ad libitum diet, leading to excessive calorie consumption and weight gain [260]. Modifying macronutrient intake in parts, such as consuming less fat or carbohydrates, is a common strategy for CR [261]. Both of these can be paired with a diet high in protein. Among these strategies, a relative increase in dietary protein intake may assist in lowering calorie intake since satiating proteins include egg whites, dairy products, and lean meat [262]. Increased consumption of specific micronutrients, such as polyphenols, may boost the antioxidant status and protect against oxidative stress [263,264]. Much evidence suggests that eating more fruits and vegetables is good for your health [265,266], maybe because of the antioxidant polyphenol concentration [267]. To help create techniques to enhance athlete performance, an additional study that simultaneously examines exercise-matched nutritional modifications is also encouraged.

This narrative review has some limitations, which should be considered. Based on limited evidence of this review about human body immune system adaptation that it can lead to alterations in recovery period immune factors, it can be highlighted that the relationship between changes in the immune system at the start of exercise and the recovery period and their effects from the CR can be comprehensively investigated. Moreover, the balance between sympathetic and parasympathetic nervous systems and their relationship with changes in CR can effectively examine changes in other factors such as sports performance, immune system and hormonal function. Therefore, not considering this vital factor in this narrative review study can be one of the major limitations. Among the other main limitations of this review is the lack of studies of the signaling pathways affecting the CR, and in this review, the annual changes and the molecular and protein mechanisms affecting the CR have not been fully investigated. In this way, in the research literature of this narrative review, a lot of effort has been made to clarify the mechanisms responsible for the difference in exercise performance during the day, but to clarify the training differences, it is necessary to examine different training models. Nutritional interventions were investigated as another effective factor in CR in this review, but more research is needed to investigate the effect of nutritional interventions more comprehensively through the careful selection of appropriate methods to measure and evaluate the effect of potential interventions. Take advantage of the CR.

## 2 Conclusion

The current narrative review provides evidence that CR is accepted as critical component of a multifaceted physiological machinery that controls and regulates many essential physiological mechanisms in most organisms, from cyanobacteria to mammals. Taking into account the individual's chronotype and using exercises at specific times of the day, it seems to be an effective way to have a major impact on physical performance, that the integration of endogenous and exogenous mechanisms has a great effect on the CR in sports performance. In addition to light, physical activity is important, which works on environmental oscillators through the master biological clock. Then they initiate many physiological functions that increase hormones, enzymes, and neurotransmitters, and through a complex network of negative feedback loops can be defined. Organism adaptation strongly depends on the internal biological clock affected by environmental conditions. Evidence shows that sleep quality and CR affect various hormones and metabolic processes. Hormones such as growth hormones, melatonin, cortisol, leptin, and ghrelin are closely related to sleep and CR.

They considered that the molecular clock has significant control over the immune system. Work on circadian inflammation to date has focused on the faster innate immune response mediated either directly by cell-intrinsic clocks or via circadian regulation by the surrounding microenvironment. However, clock proteins also significantly impact the adaptive immune response. Based on this narrative review of the available scientific literature, it appears that nutrients such as antioxidants, tryptophan-rich protein, carbohydrates, melatonin, micronutrients, and fruits can induce sleep, and there is considerable scope for further investigation of nutritional interventions. There are exercise adaptations designed to increase the quality and quantity of sleep or promote general health, sleep health, and exercise adaptations in general and athletic populations. Finally, exciting discoveries in CR research will likely benefit from the systematic examination of sex differences. The study of metabolic circadian clocks is one such area of research on CR. Some new approaches to evaluating CR deserve much attention.

## Ethics approval and consent to participate

Not Applicable.

## Funding

This research received no external funding.

## Availability of data and materials

Not Applicable.

## Author contribution statement

All authors listed have significantly contributed to the development and the writing of this article.

## Data availability statement

No data was used for the research described in the article.

## Additional information

No additional information is available for this paper.

## Declaration of competing interest

The authors declare that they have no known competing financial interests or personal relationships that could have appeared to influence the work reported in this paper.

## Abbreviations

CR: Circadian rhythms
SCN: suprachiasmatic nucleus
T: androgen testosterone
C: glucocorticoid cortisol
NFO: non-functional overtraining
Hcy: Homocysteine
Lac: blood lactate
TAS: total antioxidant status
UA: uric acid
TBIL: total bilirubin
GH: growth hormones are among the best
SWS: slow wave sleep
PVN: paraventricular nucleus
TSH: thyroid-stimulating, hormone
IGF-1: insulin-like growth factor 1
GHR: growth hormone receptor
GHRH: Growth hormone-releasing hormone
DMH: dorsal nucleus of the hypothalamus
CRH: corticotropin-releasing hormone
CCG: clock-controlled genes
5-HT: 5-hydroxytryptophan
LNAA: large neutral amino acids
GI: glycemic index
SE: sleep efficiency
TST: total sleep time
SOL: sleep onset latency
TOD: time-of-day dependent
PAL: alkaline phosphatase
GT-g: gamma-glutamyl transpeptidase
CK: creatine kinase
LDH: lactate dehydrogenase
ALAT: alanine aminotransferase
ASAT: aspartate aminotransferase
WBC: white blood cells
MO: monocytes
NE: neutrophils
LY: lymphocytes
URE: urea
CR: creatinine
GLC: glucose
TC: total cholesterol
TG: triglyceride
HDL: high-density lipoprotein
NSN: normal sleep night
PSDEN: partial sleep deprivation at the end of the night
PSDBN: partial sleep deprivation at the beginning of the night
PSG: Polysomnography
KASIP: Karolinska Athlete Screening Injury Prevention Study
NO: nitric oxide.

## References

[1] Refinetti R. (2011). Integration of biological clocks and rhythms. Compr. Physiol..

[2] Atkinson G. (2005). Diurnal variation in cycling performance: influence of warm-up. J. Sports Sci..

[3] Redlin U., Mrosovsky N. (1997). Exercise and human circadian rhythms: what we know and what we need to know. Chronobiol. Int..

[4] Reilly T. (1990). Human circadian rhythms and exercise. Crit. Rev. Biomed. Eng..

[5] Reilly T. (2007). Diurnal variation in temperature, mental and physical performance, and tasks specifically related to football (soccer). Chronobiol. Int..

[6] Bessot N. (2007). The influence of circadian rhythm on muscle activity and efficient force production during cycling at different pedal rates. J. Electromyogr. Kinesiol..

[7] Kline C.E. (2007). Circadian variation in swim performance. J. Appl. Physiol..

[8] Hastings M.H., Herzog E.D. (2004). Clock genes, oscillators, and cellular networks in the suprachiasmatic nuclei. J. Biol. Rhythm..

[9] Buijs R. (2003). Circadian and seasonal rhythms-the biological clock tunes the organs of the body: timing by hormones and the autonomic nervous system. J. Endocrinol..

[10] Guignard M.M. (1980). Circadian rhythms in plasma levels of cortisol, dehydroepiandrosterone, δ4-androstenedione, testosterone and dihydrotestosterone of healthy young men. Eur. J. Endocrinol..

[11] Hayes L.D., Bickerstaff G.F., Baker J.S. (2010). Interactions of cortisol, testosterone, and resistance training: influence of circadian rhythms. Chronobiol. Int..

[12] Bernard T. (1997). Time-of-day effects in maximal anaerobic leg exercise. Eur. J. Appl. Physiol. Occup. Physiol..

[13] Irandoust K. (2019). Effect of time-of-day-exercise in group settings on level of mood and depression of former elite male athletes. Int. J. Environ. Res. Publ. Health.

[14] McClean C., Davison G.W. (2022). Circadian clocks, redox homeostasis, and exercise: time to connect the dots?. Antioxidants.

[15] Moher D. (2009). Preferred reporting items for systematic reviews and meta-analyses: the PRISMA statement. Ann. Intern. Med..

[16] Ayala V. (2021). Influence of circadian rhythms on sports performance. Chronobiol. Int..

[17] Cajochen C., Kräuchi K., Wirz‐Justice A. (2003). Role of melatonin in the regulation of human circadian rhythms and sleep. J. Neuroendocrinol..

[18] Okamura H., Yamaguchi S., Yagita K. (2002). Molecular machinery of the circadian clock in mammals. Cell Tissue Res..

[19] Carrier J., Monk T.H. (2000). Circadian rhythms of performance: new trends. Chronobiol. Int..

[20] Wever R.A. (2013).

[21] Bellastella G. (2019). Endocrine rhythms and sport: it is time to take time into account. J. Endocrinol. Invest..

[22] Kantermann T F.S., Halle M., Schlangen L., Roenneberg T., Schmidt-Trucksäss A. (2012). The stimulating effect of bright light on physical performance depends on internal time. PLoS One.

[23] Serin Y., Tek N.A. (2019). Effect of circadian rhythm on metabolic processes and the regulation of energy balance. Ann. Nutr. Metabol..

[24] Sabzevari Rad R., Mahmoodzadeh Hosseini H., Shirvani H. (2021). Circadian rhythm effect on military physical fitness and field training: a narrative review. Sport Sci. Health.

[25] Teo W., Newton M.J., McGuigan M.R. (2011). Circadian rhythms in exercise performance: implications for hormonal and muscular adaptation. J. Sports Sci. Med..

[26] Aoyama S., Shibata S. (2020). Time-of-day-dependent physiological responses to meal and exercise. Front. Nutr..

[27] Chtourou H., Souissi N. (2012). The effect of training at a specific time of day: a review. J. Strength Condit Res..

[28] Winget C.M., DeRoshia C.W., Holley D.C. (1985). Circadian rhythms and athletic performance. Med. Sci. Sports Exerc..

[29] Starkie R.L. (1999). Effect of temperature on muscle metabolism during submaximal exercise in humans. Exp. Physiol..

[30] Smolensky M., Lamberg L. (2000).

[31] Shibata S., Tahara Y. (2014). Circadian rhythm and exercise. The Journal of Physical Fitness and Sports Medicine.

[32] Atkinson G., Speirs L. (1998). Diurnal variation in tennis service. Percept. Mot. Skills.

[33] Racinais S. (2005). Time of day influences the environmental effects on muscle force and contractility. Med. Sci. Sports Exerc..

[34] Huguet G., Touitou Y., Reinberg A. (1995). Diurnal changes in sport performance of 9-to 11-year-old school children. Chronobiol. Int..

[35] Qasrawi S.O., Pandi-Perumal S.R., BaHammam A.S. (2017). The effect of intermittent fasting during Ramadan on sleep, sleepiness, cognitive function, and circadian rhythm. Sleep Breath..

[36] Boukelia B., Sabba A., Fogarty M. (2019). The effect of zeitgeber (Fasting and Exercise) on phase advance blood glucose circadian rhythms in endurance athletes. Int. J. Soc. Sci. Humanit..

[37] Kleitman N. (1949). Biological rhythms and cycles. Physiol. Rev..

[38] Baehr E.K., Revelle W., Eastman C.I. (2000). Individual differences in the phase and amplitude of the human circadian temperature rhythm: with an emphasis on morningness–eveningness. J. Sleep Res..

[39] Grisham S.C. (1988). Diurnal variations in responses to exercise of ((MORNING types and ((EVENING types. J. Sports Med..

[40] Chelminski I. (1997). Horne and Ostberg questionnaire: a score distribution in a large sample of young adults. Pers. Indiv. Differ..

[41] Youngstedt S.D., O'Connor P.J. (1999). The influence of air travel on athletic performance. Sports Med..

[42] Sinha-Hikim I. (2002). Testosterone-induced increase in muscle size in healthy young men is associated with muscle fiber hypertrophy. Am. J. Physiol. Endocrinol. Metab..

[43] Ferrando A.A. (1998). Testosterone injection stimulates net protein synthesis but not tissue amino acid transport. Am. J. Physiol. Endocrinol. Metab..

[44] Kvorning T. (2006). Suppression of endogenous testosterone production attenuates the response to strength training: a randomized, placebo-controlled, and blinded intervention study. Am. J. Physiol. Endocrinol. Metab..

[45] Calbet J.L. (1993). Salivary steroid changes and physical performance in highly trained cyclists. Int. J. Sports Med..

[46] Tafet G.E. (2001). Correlation between cortisol level and serotonin uptake in patients with chronic stress and depression. Cognit. Affect Behav. Neurosci..

[47] Enoka R.M. (1995). Mechanisms of muscle fatigue: central factors and task dependency. J. Electromyogr. Kinesiol..

[48] Valdez P. (2019). Homeostatic and circadian regulation of cognitive performance. Biol. Rhythm. Res..

[49] Michaut A. (2003). Maximal voluntary eccentric, isometric and concentric torque recovery following a concentric isokinetic exercise. Int. J. Sports Med..

[50] Pasquet B. (2000). Muscle fatigue during concentric and eccentric contractions. Muscle Nerve: Official Journal of the American Association of Electrodiagnostic Medicine.

[51] Nicolas A. (2007). Effect of circadian rhythm of neuromuscular properties on muscle fatigue during concentric and eccentric isokinetic actions. Isokinet. Exerc. Sci..

[52] Vøllestad N.K. (1997). Measurement of human muscle fatigue. J. Neurosci. Methods.

[53] Borisenkov M. (2007). Diurnal changes in the total antioxidant activity of human saliva. Hum. Physiol..

[54] Cardona F. (2004). Periodic dip of lipidperoxidation in humans: a redox signal to synchronize peripheral circadian clocks?. Med. Hypotheses.

[55] Benot S. (1999). Physiological levels of melatonin contribute to the antioxidant capacity of human serum. J. Pineal Res..

[56] Tan D.-X. (1993). The pineal hormone melatonin inhibits DNA-adduct formation induced by the chemical carcinogen safrole in vivo. Cancer Lett..

[57] Hammouda O. (2012). High intensity exercise affects diurnal variation of some biological markers in trained subjects. Int. J. Sports Med..

[58] Deschenes M.R. (1998). Chronobiological effects on exercise performance and selected physiological responses. Eur. J. Appl. Physiol. Occup. Physiol..

[59] Dalton B., McNaughton L., Davoren B. (1997). Circadian rhythms have no effect on cycling performance. Int. J. Sports Med..

[60] Hammouda O. (2011). Diurnal variations of plasma homocysteine, total antioxidant status, and biological markers of muscle injury during repeated sprint: effect on performance and muscle fatigue—a pilot study. Chronobiol. Int..

[61] Souissi N. (2007). Effect of time of day on aerobic contribution to the 30‐s Wingate test performance. Chronobiol. Int..

[62] Hammouda O C.H., Chaouachi A., Chahed H., Bellimem H., Chamari K., Souissi N. (2013). Time-of-day effects on biochemical responses to soccer-specific endurance in elite Tunisian football players. J. Sports Sci..

[63] Waterhouse J. (2009). Changes in sleep, mood and subjective and objective responses to physical performance during the daytime in Ramadan. Biol. Rhythm. Res..

[64] Forsyth J., Reilly T. (2004). Circadian rhythms in blood lactate concentration during incremental ergometer rowing. Eur. J. Appl. Physiol..

[65] Dinges D.F. (1997). Cumulative sleepiness, mood disturbance, and psychomotor vigilance performance decrements during a week of sleep restricted to 4–5 hours per night. Sleep.

[66] Pejovic S. (2013). Effects of recovery sleep after one work week of mild sleep restriction on interleukin-6 and cortisol secretion and daytime sleepiness and performance. Am. J. Physiol. Endocrinol. Metab..

[67] Halson S.L., Jeukendrup A.E. (2004). Does overtraining exist?. Sports Med..

[68] Meeusen R. (2013). Prevention, diagnosis and treatment of the overtraining syndrome: joint consensus statement of the European college of sport science (ECSS) and the American college of sports medicine (ACSM). Eur. J. Sport Sci..

[69] Cadegiani F.A., Kater C.E. (2017). Hormonal aspects of overtraining syndrome: a systematic review. BMC Sports Science, Medicine and Rehabilitation.

[70] Chase J.D. (2017). One night of sleep restriction following heavy exercise impairs 3-km cycling time-trial performance in the morning. Appl. Physiol. Nutr. Metabol..

[71] Robson-Ansley P.J., Gleeson M., Ansley L. (2009). Fatigue management in the preparation of Olympic athletes. J. Sports Sci..

[72] Halson S.L. (2008). Nutrition, sleep and recovery. Eur. J. Sport Sci..

[73] Besedovsky L., Lange T., Born J. (2012). Sleep and immune function. Pflueg. Arch. Eur. J. Physiol..

[74] Åkerstedt T., Kecklund G., Axelsson J. (2007). Impaired sleep after bedtime stress and worries. Biol. Psychol..

[75] Petersen H. (2013). Stress vulnerability and the effects of moderate daily stress on sleep polysomnography and subjective sleepiness. J. Sleep Res..

[76] Meerlo P., Sgoifo A., Suchecki D. (2008). Restricted and disrupted sleep: effects on autonomic function, neuroendocrine stress systems and stress responsivity. Sleep Med. Rev..

[77] Hausswirth C., Louis J., Aubry A., Bonnet G., Duffield R., Le Muer, and Y. (2014). Evidence of disturbed sleep patterns and increased illness in functionally overreached endurance athletes. Med. Sci. Sports Exerc..

[78] Sargent C. (2014). The impact of training schedules on the sleep and fatigue of elite athletes. Chronobiol. Int..

[79] Roach G.D. (2013). The sleep of elite athletes at sea level and high altitude: a comparison of sea-level natives and high-altitude natives (ISA3600). Br. J. Sports Med..

[80] Lastella M. (2015). Sleep/wake behaviour of endurance cyclists before and during competition. J. Sports Sci..

[81] Sargent C., Roach G.D. (2016). Sleep duration is reduced in elite athletes following night-time competition. Chronobiol. Int..

[82] Byrd A.S., Toth A.T., Stanford F.C. (2018). Racial disparities in obesity treatment. Current obesity reports.

[83] Bruscalupi D.G.a.G. (2017). Circadian rhythms and hormonal homeostasis: pathophysiological implications. Biology Bulletin of the Russian Academy of Sciences.

[84] Patel S.A. (2016). Circadian clocks govern calorie restriction—mediated life span extension through BMAL1‐and IGF‐1‐dependent mechanisms. Faseb. J..

[85] Pietrowsky R. (1994). Effects of diurnal sleep on secretion of cortisol, luteinizing hormone, and growth hormone in man. J. Clin. Endocrinol. Metab..

[86] Weibel L., M F., Spiegel K., Gronfier C., Brandenberger G. (1997). Growth hormone secretion in night workers. Chronobiol. Int..

[87] Holl R.W. (1991). Thirty-second sampling of plasma growth hormone in man: correlation with sleep stages. J. Clin. Endocrinol. Metab..

[88] Van Cauter E. (1992). A quantitative estimation of growth hormone secretion in normal man: reproducibility and relation to sleep and time of day. J. Clin. Endocrinol. Metab..

[89] van Liempt S. (2011). Decreased nocturnal growth hormone secretion and sleep fragmentation in combat-related posttraumatic stress disorder; potential predictors of impaired memory consolidation. Psychoneuroendocrinology.

[90] Verrillo E. (2012). Effects of replacement therapy on sleep architecture in children with growth hormone deficiency. Sleep Med..

[91] Cain S.W. (2010). Sex differences in phase angle of entrainment and melatonin amplitude in humans. J. Biol. Rhythm..

[92] Gooley J.J. (2011). Exposure to room light before bedtime suppresses melatonin onset and shortens melatonin duration in humans. J. Clin. Endocrinol. Metab..

[93] R. Teclemariam-Mesbah, G.J.T.H., F. Postema, J. Wortel, and R. M. Buijs, Anatomical demonstration of the suprachiasmatic nucleus-pineal pathway*.* Comparative Neurology, 199. 406(2). 10096604

[94] Sharkey K.M., Fogg L.F., Eastman C.I. (2001). Effects of melatonin administration on daytime sleep after simulated night shift work. J. Sleep Res..

[95] Aeschbach D. (2009). Use of transdermal melatonin delivery to improve sleep maintenance during daytime. Clin. Pharmacol. Ther..

[96] Van Den Heuvel C.J., Reid K.J., Dawson D. (1997). Effect of atenolol on nocturnal sleep and temperature in young men: reversal by pharmacological doses of melatonin. Physiol. Behav..

[97] Scheer F. (2006). Reduced sleep efficiency in cervical spinal cord injury; association with abolished night time melatonin secretion. Spinal Cord.

[98] Wyatt J.K. (2006). Sleep-facilitating effect of exogenous melatonin in healthy young men and women is circadian-phase dependent. Sleep.

[99] Burgess H.J. (2010). Human phase response curves to three days of daily melatonin: 0.5 mg versus 3.0 mg. J. Clin. Endocrinol. Metab..

[100] Sack R.L. (2000). Entrainment of free-running circadian rhythms by melatonin in blind people. N. Engl. J. Med..

[101] Allan J.S., Czeisler C. (1994). Persistence of the circadian thyrotropin rhythm under constant conditions and after light-induced shifts of circadian phase. J. Clin. Endocrinol. Metab..

[102] Wehr T.A. (1993). Conservation of photoperiod-responsive mechanisms in humans. Am. J. Physiol. Regul. Integr. Comp. Physiol..

[103] Goichot B. (1992). Nocturnal plasma thyrotropin variations are related to slow‐wave sleep. J. Sleep Res..

[104] Gronfier C. (1995). Temporal link between plasma thyrotropin levels and electroencephalographic activity in man. Neurosci. Lett..

[105] Wehr T., Aeschbach D., Duncan W. (2001). Evidence for a biological dawn and dusk in the human circadian timing system. J. Physiol..

[106] Scheer F.A. (2009). Adverse metabolic and cardiovascular consequences of circadian misalignment. Proc. Natl. Acad. Sci. USA.

[107] Windle R. (1998). Ultradian rhythm of basal corticosterone release in the female rat: dynamic interaction with the response to acute stress. Endocrinology.

[108] Young E.A., Abelson J., Lightman S.L. (2004). Cortisol pulsatility and its role in stress regulation and health. Front. Neuroendocrinol..

[109] Buijs R.M. (1999). Anatomical and functional demonstration of a multisynaptic suprachiasmatic nucleus adrenal (cortex) pathway. Eur. J. Neurosci..

[110] Steiger A. (2002). Sleep and the hypothalamo–pituitary–adrenocortical system. Sleep Med. Rev..

[111] Esler W.P. (2007). Small-molecule ghrelin receptor antagonists improve glucose tolerance, suppress appetite, and promote weight loss. Endocrinology.

[112] Clement K. (1998). A mutation in the human leptin receptor gene causes obesity and pituitary dysfunction. Nature.

[113] Kluge M. (2010). Ghrelin increases slow wave sleep and stage 2 sleep and decreases stage 1 sleep and REM sleep in elderly men but does not affect sleep in elderly women. Psychoneuroendocrinology.

[114] Dzaja A. (2004). Sleep enhances nocturnal plasma ghrelin levels in healthy subjects. Am. J. Physiol. Endocrinol. Metab..

[115] Schuessler P. (2005). Nocturnal ghrelin levels–relationship to sleep EEG, the levels of growth hormone, ACTH and cortisol–and gender differences. J. Sleep Res..

[116] Shea S.A. (2005). Independent circadian and sleep/wake regulation of adipokines and glucose in humans. J. Clin. Endocrinol. Metab..

[117] Dehghani S., Mehri S., Hosseinzadeh H. (2019). The effects of Crataegus pinnatifida (Chinese hawthorn) on metabolic syndrome: a review. Iranian journal of basic medical sciences.

[118] Hastings M.H., Maywood E.S., Brancaccio M. (2018). Generation of circadian rhythms in the suprachiasmatic nucleus. Nat. Rev. Neurosci..

[119] Takahashi J.S. (2017). Transcriptional architecture of the mammalian circadian clock. Nat. Rev. Genet..

[120] Westwood M.L. (2019). The evolutionary ecology of circadian rhythms in infection. Nature ecology & evolution.

[121] Man K., Loudon A., Chawla A. (2016). Immunity around the clock. Science.

[122] Scheiermann C., Kunisaki Y., Frenette P.S. (2013). Circadian control of the immune system. Nat. Rev. Immunol..

[123] Pacheco-Bernal I., Becerril-Pérez F., Aguilar-Arnal L. (2019). Circadian rhythms in the three-dimensional genome: implications of chromatin interactions for cyclic transcription. Clin. Epigenet..

[124] Haus E., Smolensky M.H. (1999). Biologic rhythms in the immune system. Chronobiol. Int..

[125] Dimitrov S. (2009). Cortisol and epinephrine control opposing circadian rhythms in T cell subsets. Blood, the Journal of the American Society of Hematology.

[126] Scheiermann C. (2012). Adrenergic nerves govern circadian leukocyte recruitment to tissues. Immunity.

[127] Hawley J.A. (2014). Integrative biology of exercise. Cell.

[128] Thomas J.M. (2020). Circadian rhythm phase shifts caused by timed exercise vary with chronotype. JCI insight.

[129] Song Y. (2018). Circadian rhythm gene expression and daily melatonin levels vary in athletes and sedentary males. Biol. Rhythm. Res..

[130] Weinert D., Gubin D. (2022). The impact of physical activity on the circadian system: benefits for health, performance and Wellbeing. Appl. Sci..

[131] Waterhouse J. (2005). The circadian rhythm of core temperature: origin and some implications for exercise performance. Chronobiol. Int..

[132] Ezagouri S. (2019). Physiological and molecular dissection of daily variance in exercise capacity. Cell Metabol..

[133] Atkinson G., Reilly T. (1996). Circadian variation in sports performance. Sports Med..

[134] Ammar A. (2016). Relationship between biomarkers of muscle damage and redox status in response to a weightlifting training session: effect of time-of-day. Acta Physiol. Hung..

[135] Ammar A C.H., Trabelsi K., Padulo J., Turki M., El Abed K., Hoekelmann A., Hakim A. (2015). Temporal specificity of training: intra-day effects on biochemical responses and Olympic weightlifting performances. J. Sports Sci..

[136] Chtourou H. (2012). Diurnal variations in physical performances related to football in young soccer players. Asian J. Sports Med..

[137] Chtourou H. (2018). Diurnal variation of short-term repetitive maximal performance and psychological variables in elite judo athletes. Front. Physiol..

[138] Ammar A. (2015). Acute and delayed responses of C-reactive protein, malondialdehyde and antioxidant markers after resistance training session in elite weightlifters: effect of time of day. Chronobiol. Int..

[139] Henst R.H. (2015). A chronotype comparison of South African and Dutch marathon runners: the role of scheduled race start times and effects on performance. Chronobiol. Int..

[140] di Cagno A. (2013). Time of day–effects on motor coordination and reactive strength in elite athletes and untrained adolescents. J. Sports Sci. Med..

[141] Copenhaver E.A., Diamond A.B. (2017). The value of sleep on athletic performance, injury, and recovery in the young athlete. Pediatr. Ann..

[142] Ruddick‐Collins L. (2018). The Big Breakfast Study: chrono‐nutrition influence on energy expenditure and bodyweight. Nutr. Bull..

[143] Ruddick‐Collins L.C., Morgan P.J., Johnstone A.M. (2020). Mealtime: a circadian disruptor and determinant of energy balance?. J. Neuroendocrinol..

[144] Pickel L., Sung H.-K. (2020). Feeding rhythms and the circadian regulation of metabolism. Front. Nutr..

[145] Tahara Y., Shibata S. (2014). Chrono-biology, chrono-pharmacology, and chrono-nutrition. J. Pharmacol. Sci..

[146] Saper C.B., Scammell T.E., Lu J. (2005). Hypothalamic regulation of sleep and circadian rhythms. Nature.

[147] Halson S.L. (2014). Sleep in elite athletes and nutritional interventions to enhance sleep. Sports Med..

[148] Silber B., Schmitt J. (2010). Effects of tryptophan loading on human cognition, mood, and sleep. Neurosci. Biobehav. Rev..

[149] Afaghi A., O'Connor H., Chow C.M. (2007). High-glycemic-index carbohydrate meals shorten sleep onset. Am. J. Clin. Nutr..

[150] Peuhkuri K., Sihvola N., Korpela R. (2012). Diet promotes sleep duration and quality. Nutr. Res. (N.Y.).

[151] Golem D.L. (2014). An integrative review of sleep for nutrition professionals. Adv. Nutr..

[152] Ordóñez F.M. (2017). Sleep improvement in athletes: use of nutritional supplements. Am. J. Sports Med..

[153] Grandner M.A. (2014). Sleep symptoms associated with intake of specific dietary nutrients. J. Sleep Res..

[154] Porter J., Horne J. (1981). Bed-time food supplements and sleep: effects of different carbohydrate levels. Electroencephalogr. Clin. Neurophysiol..

[155] Lin H.-H. (2011). Effect of kiwifruit consumption on sleep quality in adults with sleep problems. Asia Pac. J. Clin. Nutr..

[156] Valtonen M. (2005). Effect of melatonin-rich night-time milk on sleep and activity in elderly institutionalized subjects. Nord. J. Psychiatr..

[157] Garrido M. (2010). Jerte Valley cherry-enriched diets improve nocturnal rest and increase 6-sulfatoxymelatonin and total antioxidant capacity in the urine of middle-aged and elderly humans. Journals of Gerontology Series A: Biomedical Sciences and Medical Sciences.

[158] Howatson G. (2012). Effect of tart cherry juice (Prunus cerasus) on melatonin levels and enhanced sleep quality. Eur. J. Nutr..

[159] Thomas D.T.E., K A., Burke L.M. (2016). American college of sports medicine joint position Statement.Nutrition and athletic performance. Med. Sci. Sports Exerc..

[160] Close G.L. (2016). New strategies in sport nutrition to increase exercise performance. Free Radical Biol. Med..

[161] Jeukendrup A.E. (2017). Periodized nutrition for athletes. Sports Med..

[162] Roehrs T., Roth T. (2001). Sleep, sleepiness, and alcohol use. Alcohol Res. Health.

[163] Leonardo-Mendonça R.C. (2015). The benefits of four weeks of melatonin treatment on circadian patterns in resistance-trained athletes. Chronobiol. Int..

[164] Cheikh M. (2018). Melatonin ingestion after exhaustive late-evening exercise improves sleep quality and quantity, and short-term performances in teenage athletes. Chronobiol. Int..

[165] Atkinson G. (2001). Are there hangover-effects on physical performance when melatonin is ingested by athletes before nocturnal sleep?. Int. J. Sports Med..

[166] Ghattassi K. (2016). Morning melatonin ingestion and diurnal variation of short-term maximal performances in soccer players. Acta Physiol. Hung..

[167] Hudson C. (2005). Protein source tryptophan versus pharmaceutical grade tryptophan as an efficacious treatment for chronic insomnia. Nutr. Neurosci..

[168] Rondanelli M. (2011). The effect of melatonin, magnesium, and zinc on primary insomnia in long‐term care facility residents in Italy: a double‐blind, placebo‐controlled clinical trial. J. Am. Geriatr. Soc..

[169] Pires M.L.N. (2001). Acute effects of low doses of melatonin on the sleep of young healthy subjects. J. Pineal Res..

[170] Markus C.R. (2005). Evening intake of α-lactalbumin increases plasma tryptophan availability and improves morning alertness and brain measures of attention. Am. J. Clin. Nutr..

[171] López-Flores M. (2018). Effects of melatonin on sports performance: a systematic review. Journal of Exercise Physiology Online.

[172] Meoli A.L. (2005). Oral nonprescription treatment for insomnia: an evaluation of products with limited evidence. J. Clin. Sleep Med..

[173] Ammar A., Chtourou H., Souissi N. (2017). Effect of time-of-day on biochemical markers in response to physical exercise. J. Strength Condit Res..

[174] Joubert L.M., Manore M.M. (2006). Exercise, nutrition, and homocysteine. Int. J. Sport Nutr. Exerc. Metabol..

[175] Bazyar Y. (2015). An Animal Model Of Alzheimer’s Disease.

[176] Kanabrocki E. (1988). Ten-year-replicated circadian profiles for 36 physiological, serological and urinary variables in healthy men. Chronobiol. Int..

[177] Rivera-Coll A., Fuentes-Arderiu X., Díez-Noguera A. (1993). Circadian rhythms of serum concentrations of 12 enzymes of clinical interest. Chronobiol. Int..

[178] Mejri M.A. (2015). One night of partial sleep deprivation affects biomarkers of cardiac damage, but not cardiovascular and lipid profiles, in young athletes. Biol. Rhythm. Res..

[179] Knutson K.L. (2007). The metabolic consequences of sleep deprivation. Sleep Med. Rev..

[180] O'Neill J.S., Reddy A.B. (2011). Circadian clocks in human red blood cells. Nature.

[181] Abedelmalek S. (2013). Effects of partial sleep deprivation on proinflammatory cytokines, growth hormone, and steroid hormone concentrations during repeated brief sprint interval exercise. Chronobiol. Int..

[182] Rae D.E. (2017). One night of partial sleep deprivation impairs recovery from a single exercise training session. Eur. J. Appl. Physiol..

[183] Alzoubi K.H. (2013). The combined effect of sleep deprivation and Western diet on spatial learning and memory: role of BDNF and oxidative stress. J. Mol. Neurosci..

[184] Savic-Radojevic A. (2015). Effect of hyperglycemia and hyperinsulinemia on glutathione peroxidase activity in non-obese women with polycystic ovary syndrome. Hormones (Basel).

[185] Smolensky M.H., Dalonzo G.E. (1993). Medical chronobiology concepts and appications. Am, Rev, Respir.

[186] Melhim A.F. (1993). Investigation of circadian rhythms in peak power and mean power of female physical education students. Int. J. Sports Med..

[187] Borbély A.A. (2016). The two‐process model of sleep regulation: a reappraisal. J. Sleep Res..

[188] Borbély A.A. (1982). A two process model of sleep regulation. Hum. Neurobiol..

[189] Czeisler C.A. (1999). Stability, precision, and near-24-hour period of the human circadian pacemaker. Science.

[190] Irwin M.R., Olmstead R., Carroll J.E. (2016). Sleep disturbance, sleep duration, and inflammation: a systematic review and meta-analysis of cohort studies and experimental sleep deprivation. Biol. Psychiatr..

[191] Frank M.G. (2006). The mystery of sleep function: current perspectives and future directions. Rev. Neurosci..

[192] Irwin M.R. (2015). Why sleep is important for health: a psychoneuroimmunology perspective. Annu. Rev. Psychol..

[193] Tuomilehto H. (2017). Sleep of professional athletes: underexploited potential to improve health and performance. J. Sports Sci..

[194] Erlacher D. (2011). Sleep habits in German athletes before important competitions or games. J. Sports Sci..

[195] Milewski M.D. (2014). Chronic lack of sleep is associated with increased sports injuries in adolescent athletes. J. Pediatr. Orthop..

[196] Von Rosen P. (2017). Too little sleep and an unhealthy diet could increase the risk of sustaining a new injury in adolescent elite athletes. Scand. J. Med. Sci. Sports.

[197] Meney I. (1998). The effect of one night's sleep deprivation on temperature, mood, and physical performance in subjects with different amounts of habitual physical activity. Chronobiol. Int..

[198] de Zwart B.C. (1993). After-effects of night work on physical performance capacity and sleep quality in relation to age. Int. Arch. Occup. Environ. Health.

[199] Martin B.J. (1981). Effect of sleep deprivation on tolerance of prolonged exercise. Eur. J. Appl. Physiol. Occup. Physiol..

[200] Souissi N. (2003). Effects of one night's sleep deprivation on anaerobic performance the following day. Eur. J. Appl. Physiol..

[201] Souissi N. (2008). Effect of time of day and partial sleep deprivation on short‐term, high‐power output. Chronobiol. Int..

[202] Bougard C., Davenne D. (2012). Effects of sleep deprivation and time-of-day on selected physical abilities in off-road motorcycle riders. Eur. J. Appl. Physiol..

[203] Mougin F. (1996). Effects of a selective sleep deprivation on subsequent anaerobic performance. Int. J. Sports Med..

[204] Rodgers C. (1995). Sleep deprivation: effects on work capacity, self-paced walking, contractile properties and perceived exertion. Sleep.

[205] Reilly T., Piercy M. (1994). The effect of partial sleep deprivation on weight-lifting performance. Ergonomics.

[206] Waterhouse J. (2007). The role of a short post-lunch nap in improving cognitive, motor, and sprint performance in participants with partial sleep deprivation. J. Sports Sci..

[207] Reilly T., Atkinson G., Waterhouse J.M. (1997).

[208] Mah C.D. (2011). The effects of sleep extension on the athletic performance of collegiate basketball players. Sleep.

[209] Blumert P.A. (2007). The acute effects of twenty-four hours of sleep loss on the performance of nationalcaliber male collegiate weightlifters. J. Strength Condit Res..

[210] Athey A.B., Ross M.J. (2019).

[211] Mah C.D. (2018). Poor sleep quality and insufficient sleep of a collegiate student-athlete population. Sleep health.

[212] Schwartz J., Simon R.D. (2015). Sleep extension improves serving accuracy: a study with college varsity tennis players. Physiol. Behav..

[213] Services, U.D.o.H.a.H. (2015). Sleep health objectives. Mar.

[214] Lemola S. (2015). Adolescents' electronic media use at night, sleep disturbance, and depressive symptoms in the smartphone age. J. Youth Adolesc..

[215] Pimputkar S. (2009). Prospects for LED lighting. Nat. Photonics.

[216] Gringras P. (2015). Bigger, brighter, bluer-better? Current light-emitting devices–adverse sleep properties and preventative strategies. Front. Public Health.

[217] Cajochen C. (2011). Evening exposure to a light-emitting diodes (LED)-backlit computer screen affects circadian physiology and cognitive performance. J. Appl. Physiol..

[218] Munch M. (2006). Wavelength-dependent effects of evening light exposure on sleep architecture and sleep EEG power density in men. Am. J. Physiol. Regul. Integr. Comp. Physiol..

[219] Hannibal J. (2017). Melanopsin expressing human retinal ganglion cells: subtypes, distribution, and intraretinal connectivity. J. Comp. Neurol..

[220] Kern S. (2008). Glucose metabolic changes in the prefrontal cortex are associated with HPA axis response to a psychosocial stressor. Psychoneuroendocrinology.

[221] Schmidt C. (2018). Light exposure via a head‐mounted device suppresses melatonin and improves vigilant attention without affecting cortisol and comfort. PsyCh J..

[222] Leproult R. (2001). Transition from dim to bright light in the morning induces an immediate elevation of cortisol levels. J. Clin. Endocrinol. Metab..

[223] Chang A.-M. (2015). Evening use of light-emitting eReaders negatively affects sleep, circadian timing, and next-morning alertness. Proc. Natl. Acad. Sci. USA.

[224] Knufinke M. (2019). Restricting short-wavelength light in the evening to improve sleep in recreational athletes–A pilot study. Eur. J. Sport Sci..

[225] Edwards B.J., Lindsay K., Waterhouse J. (2005). Effect of time of day on the accuracy and consistency of the badminton serve. Ergonomics.

[226] Reilly T., Down A. (1986).

[227] Gauthier A. (1996). Diurnal rhythm of the muscular performance of elbow flexors during isometric contractions. Chronobiol. Int..

[228] Gauthier A. (1997). Orcadian rhythm in the torque developed by elbow flexors during isometric contraction effect of sampling schedules. Chronobiol. Int..

[229] Gauthier A. (2001). Time of day effects on isometric and isokinetic torque developed during elbow flexion in humans. Eur. J. Appl. Physiol..

[230] Souissi N. (2004). Circadian rhythms in two types of anaerobic cycle leg exercise: force-velocity and 30-s Wingate tests. Int. J. Sports Med..

[231] Reilly T., Down A. (1992). Investigation of circadian rhythms in anaerobic power and capacity of the legs. J. Sports Med. Phys. Fit..

[232] Javierre C. (1996). Influence of sleep and meal schedules on performance peaks in competitive sprinters. Int. J. Sports Med..

[233] Pilcher J.J., Huffcutt A.I. (1996). Effects of sleep deprivation on performance: a meta-analysis. Sleep.

[234] Paul K.N., Turek F.W., Kryger M.H. (2008). Influence of sex on sleep regulatory mechanisms. J. Wom. Health.

[235] Thordstein M.L.N. (2006). Sex differences in electrocortical activity in human neonates. Neuroreport.

[236] Menna-Barreto L. (1989). The sleep/wake cycle in 4-to 14-month old children: general aspects and sex differences. Brazilian Journal of Medical and Biological Research= Revista Brasileira de Pesquisas Medicas e Biologicas.

[237] Nagy E. (2001). Gender-related physiologic differences in human neonates and the greater vulnerability of males to developmental brain disorders. J. Gender-Specific Med. (JGSM): JGSM: the official journal of the Partnership for Women's Health at Columbia.

[238] Roenneberg T. (2004). A marker for the end of adolescence. Curr. Biol..

[239] Hagenauer M.H., Lee T.M. (2012). The neuroendocrine control of the circadian system: adolescent chronotype. Front. Neuroendocrinol..

[240] Sadeh A. (2009). Sleep and the transition to adolescence: a longitudinal study. Sleep.

[241] Manber R., Baker F., Gress J. (2006). Sex differences in sleep and sleep disorders: a focus on women's sleep. Int J Sleep Disorders.

[242] Zhang B., Wing Y.-K. (2006). Sex differences in insomnia: a meta-analysis. Sleep.

[243] Buysse D.J. (2008). EEG spectral analysis in primary insomnia: NREM period effects and sex differences. Sleep.

[244] Mong J.A. (2011). Sleep, rhythms, and the endocrine brain: influence of sex and gonadal hormones. J. Neurosci..

[245] Armitage R. (2001). Sex differences in slow-wave activity in response to sleep deprivation. Sleep Res. Online.

[246] Dijk D.J., Beersma D.G., Bloem G.M. (1989). Sex differences in the sleep EEG of young adults: visual scoring and spectral analysis. Sleep.

[247] Ehlers C., Kupfer D. (1997). Slow‐wave sleep: do young adult men and women age differently?. J. Sleep Res..

[248] Paul K.N. (2006). Diurnal sex differences in the sleep-wake cycle of mice are dependent on gonadal function. Sleep.

[249] Reid K.J. (2019). Assessment of circadian rhythms. Neurol. Clin..

[250] Touati S. (2015). Exercise training protects against atherosclerotic risk factors through vascular NADPH oxidase, extracellular signal‐regulated kinase 1/2 and stress‐activated protein kinase/c‐J un N‐terminal kinase downregulation in obese rats. Clin. Exp. Pharmacol. Physiol..

[251] Ramezanipour M. (2014). The effect of weight reduction on antioxidant enzymes and their association with dietary intake of vitamins A, C and E. Arquivos Brasileiros Endocrinol. Metabol..

[252] Bougoulia M., Triantos A., Koliakos G. (2006). Plasma interleukin-6 levels, glutathione peroxidase and isoprostane in obese women before and after weight loss. Association with cardiovascular risk factors. Hormones-Athens-.

[253] Wycherley T. (2008). Effect of caloric restriction with and without exercise training on oxidative stress and endothelial function in obese subjects with type 2 diabetes. Diabetes Obes. Metabol..

[254] Bloomer R.J. (2010). Postprandial oxidative stress in response to dextrose and lipid meals of differing size. Lipids Health Dis..

[255] Liu Y., Fiskum G., Schubert D. (2002). Generation of reactive oxygen species by the mitochondrial electron transport chain. J. Neurochem..

[256] Bloomer R.J. (2011). A 21 day Daniel Fast improves selected biomarkers of antioxidant status and oxidative stress in men and women. Nutr. Metab..

[257] McCarthy C.G. (2013). High-fat feeding, but not strenuous exercise, increases blood oxidative stress in trained men. Appl. Physiol. Nutr. Metabol..

[258] Goldberg E.L. (2015). Lifespan‐extending caloric restriction or m TOR inhibition impair adaptive immunity of old mice by distinct mechanisms. Aging Cell.

[259] Jové M. (2014). Caloric restriction reveals a metabolomic and lipidomic signature in liver of male mice. Aging Cell.

[260] Sohal R.S., Forster M.J. (2014). Caloric restriction and the aging process: a critique. Free Radic. Biol. Med..

[261] Noakes T.D. (2013). Low-carbohydrate and high-fat intake can manage obesity and associated conditions: occasional survey. S. Afr. Med. J..

[262] Kremsdorf R.A. (2013). Effects of a high-protein diet on regulation of phosphorus homeostasis. J. Clin. Endocrinol. Metab..

[263] Mennen L.I. (2004). Consumption of foods rich in flavonoids is related to a decreased cardiovascular risk in apparently healthy French women. J. Nutr..

[264] Urquiaga I., Leighton F. (2000). Plant polyphenol antioxidants and oxidative stress. Biol. Res..

[265] Liu R.H. (2003). Health benefits of fruit and vegetables are from additive and synergistic combinations of phytochemicals. Am. J. Clin. Nutr..

[266] Jiang Y. (2014). Cruciferous vegetable intake is inversely correlated with circulating levels of proinflammatory markers in women. J. Acad. Nutr. Diet..

[267] Feskanich D. (2000). Prospective study of fruit and vegetable consumption and risk of lung cancer among men and women. J. Natl. Cancer Inst..

